# Prospective comparison of two models of integrating early infant male circumcision with maternal child health services in Kenya: The Mtoto Msafi Mbili Study

**DOI:** 10.1371/journal.pone.0184170

**Published:** 2017-09-07

**Authors:** Robert C. Bailey, Fredrick Adera, Mary Ellen Mackesy-Amiti, Timothy Adipo, Sherry K. Nordstrom, Supriya D. Mehta, Walter Jaoko, F. L. Fredrik G. Langi, Walter Obiero, Edmon Obat, Fredrick O. Otieno, Marisa R. Young

**Affiliations:** 1 Division of Epidemiology and Biostatistics, School of Public Health, University of Illinois at Chicago, Chicago, IL, United States of America; 2 Nyanza Reproductive Health Society, Kisumu, Kenya; 3 Advocate Medical Group, Advocate Christ Medical Center, Oak Lawn, IL, United States of America; 4 Department of Medical Microbiology, University of Nairobi, Nairobi, Kenya; 5 Division of Health Policy and Administration, School of Public Health, University of Illinois at Chicago, Chicago, IL, United States of America; 6 Department of Gynecology and Obstetrics, Emory University, Atlanta, GA, United States of America; Johns Hopkins University Bloomberg School of Public Health, UNITED STATES

## Abstract

As countries scale up adult voluntary medical male circumcision (VMMC) for HIV prevention, they are looking ahead to long term sustainable strategies, including introduction of early infant male circumcision (EIMC). To address the lack of evidence regarding introduction of EIMC services in sub-Saharan African settings, we conducted a simultaneous, prospective comparison of two models of EIMC service delivery in Homa Bay County, Kenya. In one division a standard delivery package (SDP) was introduced and included health facility-based provision of EIMC services with community engagement for client referral versus in a different division a standard package plus (SDPplus) that included community-delivered EIMC services. Babies 1–60 days old were eligible for EIMC. A representative sample of mothers and fathers of baby boys at 16 health facilities was surveyed. We examined differences between mothers and fathers in the SDP and SDPplus divisions and identified factors associated with EIMC uptake. We report adjusted prevalence ratios (aPR). Of 1660 mothers interviewed, 1501 (89%) gave approval to contact the father, and 1259 fathers (84%) were interviewed. The proportion of babies circumcised was slightly greater in the SDPplus division than the SDP division (27.3% vs 23.7%), but the difference was not significant (p = 0.08). In adjusted analyses, however, the prevalence of babies being circumcised was greater in the SDPplus division (aPR = 1.23, 95% CI:1.04–1.45) and the factors associated with a baby being circumcised were the mother having received information about EIMC (during pregnancy, aPR = 4.81, 95% CI: 2.21–3.42), having discussed circumcision with the father if married or cohabiting (aPR = 5.39, 95% CI: 3.31–8.80) or being single (aPR = 5.67, 95% CI: 3.31–9.69), perceiving herself to be living with HIV (aPR = 1.39, 95% CI: 1.15–1.67), or having a post-secondary education (aPR = 1.33, 95% CI: 1.04–1.69), and the father being Muslim (aPR = 1.85, 95% CI: 1.29–2.65) or circumcised (aPR = 1.34, 95% CI: 1.13–1.59). The median age of 2117 babies circumcised was 8 days (IQR: 1–36), and the median weight was 3.6 kg (IQR: 3.2–4.4). There were 6 moderate adverse events (AEs) (0.28%); 5 severe AEs (0.24%), all involving an injury to the glans penis, requiring hospitalization and corrective surgery; and one death probably related to the procedure. There were no AEs among the 365 procedures performed outside health facilities. Information and education campaigns must reach members of the general population, especially men and fathers, who are influential to the EIMC decision. Serious AEs using the Mogen clamp are rare, but do occur and require efficient, reliable emergency back-up. Our results can assist countries considering scale-up of EIMC services for HIV prevention as their adult VMMC programs mature.

## Introduction

Numerous observational studies and three randomized controlled trials have shown male circumcision to be approximately 60% effective in reducing HIV acquisition in heterosexual men in sub-Saharan Africa [1–4]. WHO/UNAIDS urges male circumcision be offered as one component of a comprehensive HIV prevention package and recommends that countries consider early infant male circumcision (EIMC) as a long-term sustainable HIV prevention strategy [5]. Large scale EIMC would represent a transition from managing the HIV epidemic as an emergency towards a focus on sustainable, long-term solutions to this major global health challenge. Recognizing this, the Kenyan Government’s national strategy to scale up voluntary medical male circumcision includes plans to transition from adolescent and adult male circumcision (AMC) to predominantly EIMC [6].

To date, there have been very few studies attempting to introduce EIMC in communities in East and southern Africa. Although evidence is lacking, many have stated that EIMC in Africa would have numerous advantages over AMC. Compared to AMC, circumcision of an infant is thought to be associated with fewer adverse events, to be faster and less technically challenging, to cost less, to be easier to care for post-operatively, and likely to reduce chances of sexual disinhibition or risk compensation [7–9]. It is well established that benefits to boys circumcised in infancy include reduction in urinary tract infections and avoidance of phimosis, paraphimosis and other conditions affecting the foreskin [10,11]. The same health benefits afforded circumcised men later in life accrue to those circumcised in infancy, including reduction in genital ulcer disease, reduction in oncogenetic HPV prevalence and incidence, lower rates of penile cancer, and reduction in risk of HIV acquisition, among others [12]. Benefits to female sexual partners of circumcised men include reduced risk of Bacterial vaginosis, *Trichomonas vaginalis*, HPV infection and cervical cancer [12–15]. However, despite the many potential advantages of circumcising infants, possible drawbacks include lack of familiarity with EIMC in countries in East and southern Africa and the lengthy interval between the procedure and impact on the HIV epidemic [16–18]. Many have stated that EIMC can be seamlessly and safely integrated with existing maternal neonatal child health (MNCH) programs [6–8], but this could prove challenging in resource poor settings where programs are already stretched to achieve MNCH targets. Moreover, as our previous studies in Kenya have shown, approximately half of mothers do not deliver in health facilities, and involvement of fathers is crucial to the EIMC decision [16,19]. These factors make significant uptake and safe, efficient implementation of a facility-based EIMC program extremely challenging.

To address the lack of evidence regarding introduction of EIMC services in sub-Saharan African settings, we conducted a simultaneous, prospective comparison of two models of EIMC service delivery: a standard delivery package (SDP) that included health facility-based provision of EIMC services with community engagement for client referral versus a standard package plus (SDPplus) that included community-delivered IMC services. We assessed differences between the two packages in EIMC uptake, adverse event rates, knowledge of EIMC and various parental behavioral and demographic characteristics.

## Methods

### Study area and population

The study area lies within Homa Bay County in western Kenya adjacent to Lake Victoria. The predominant ethnic group is Luo, a southern Sudan-speaking people who do not traditionally practice circumcision. Adult HIV prevalence in Homa Bay County is the highest in Kenya at 27.1% compared to 5.6% for the country overall [20]. Homa Bay County has six sub-counties divided into Divisions. This study was conducted in Kasipul Division of Rachuonyo South and Karachuonyo West Division of Rachuonyo North. These division borders are separated by approximately 10 kilometers and, since most people do not own cars and travel by foot or bicycle for vaccination or other MNCH services, contamination across divisions is unlikely. Population sizes are approximately 164,000 for Kasipul Division (the SDPplus Division) and 90,000 for Karachuonyo West Division (the SDP Division). Both divisions contain one large trading center with the majority of the population in both districts residing in rural areas and practicing either farming or fishing. Infant male circumcision is little-known and little-practiced in East and southern Africa generally and this was true in Homa Bay County before the onset of this project. EIMC was not part of routine antenatal education and was not normally discussed post-partum nor during immunization visits. This was evident during the baseline assessment of EIMC in the two study divisions. Among 613 babies brought to health facilities for their first Oral Polio Vaccine (OPV-1) at six weeks post-partum, none were circumcised [19].

The study was a simultaneous, prospective comparison of two models of EIMC service delivery—the Standard Delivery Package (SDP) and the Standard Delivery Package Plus (SDPplus)—comparing rates and safety of EIMC, and barriers and facilitators of EIMC uptake between packages before and after implementation of EIMC services. Both models employed a community-based strategy for disseminating information about EIMC to adult men and perinatal women utilizing the existing structure of community health workers (CHWs) and health educators who are attached to every health facility. The community-based education strategy, facility health talks and facility-based provision of EIMC surgery form the SDP. The SDPplus included the same activities as in the SDP, with the added component of training retired and unemployed nurse-midwives residing in the community to identify pregnant women, to supplement the EIMC education provided by the CHWs, to actively engage fathers pre-delivery, and to perform EIMCs in the community for those not seeking the services in a health facility. We hypothesized that the SDPplus package of services would result in greater uptake of EIMC and have the same level of safety as the SDP model, measured by adverse events (AEs).

Both the SDP and SDPplus models employed a comprehensive community strategy for recruitment of infants for EIMC. Under both models, MOH providers at four facilities were trained to provide comprehensive IMC services. Since most government facilities suffer from understaffing, to ensure adequate human resource capacity to provide EIMC services, we used an existing MOH model, termed “direct hire,” to engage an additional clinical staff person (nurse or clinical officer) who worked as an MOH employee at the health facility. At the two largest health facilities (Rachuonyo South and Rachuonyo North sub-County Hospitals), all nurses and clinical officers on the maternity ward and in the MCH clinic were trained in EIMC service delivery. At smaller facilities, at least one staff in addition to the direct hire was similarly trained. The responsibilities of the direct hire staff were not solely performance of EIMC services, but were to contribute to all activities at their workstation, providing the time necessary for any of the trained qualified staff to perform EIMCs

Various strategies were employed to sensitize the communities of both divisions about the risks, benefits and availability of EIMC. A sensitization campaign targeting village elders, chiefs, and men who had chosen MC for themselves was employed to reach men. Educational talks were delivered in chiefs’ barazas (meetings), at football matches, in markets, in popular bars and restaurants, and at other venues where men congregate. The educational fliers were distributed and men were encouraged to tell friends about the service. At the health facilities, MNCH staff were trained on the benefits and risks of EIMC and how to provide information about EIMC in a culturally appropriate and supportive manner. Mothers accessing antenatal, delivery, and postnatal services were given informational talks about EIMC in groups and, as needed, individually. An already existing network of CHWs attached to health facilities were trained to provide information at the household level on the benefits and risks of EIMC. In Kenya, each CHW is responsible for between 20 and 100 homes. The role of the CHW is to visit each home at least once per month and serve as a link between the community and the health facility, and their functions include recording the number of pregnant women, the number of recent deliveries and the vaccine coverage of any children in every home. CHWs in both divisions were trained to counsel pregnant and postnatal mothers and their male partners at home about EIMC. The CHWs were provided with referral cards, stamped with the name/ID number of the CHW, and when parents brought their child with the referral card to the health facility for circumcision, the CHW was compensated 200 Kenyan shillings (approximately $2.00US) for his or her time and effort. A similar system of compensation to CHWs for recruiting adult MC clients was already in place.

Additional compensation was provided to the health facility to cover the costs of water, electricity, cleaning, and any other incidental costs. This was in the amount of 2000 Kenya shillings ($20.00US) for every 10 EIMC procedures performed on-site.

The only difference between the SDP division and the SDPplus division was the addition of Domiciliary Midwives (DMs) in the latter. DMs were recruited by identifying retired or unemployed health workers living in the community. This is a cadre of health workers that have been targeted by the Kenyan MOH to be used by health facilities to augment their community programs, including skilled deliveries outside health facilities, because of their high level of training, under-utilization, and close connection to the community. Beyond providing EIMC services, DMs were trained to deliver a comprehensive package of MNCH services consistent with Kenya Essential Package for Health. DMs were paid a low base salary of 22000 KSH ($220US) per month and provided modest monetary compensation (250 KSH; $2.50US) for conducting EIMC services in the home or, similar to a CHW, 200 KSH for referring parents to the health facility for EIMC services.

Circumcisions were performed by providers who had completed a minimum of 10 procedures under supervision with additional training as needed. Circumcisions were performed employing the Mogen clamp as described in the WHO “Manual for Early Infant Male Circumcision under Local Anaesthesia” [9]. At least one parent was required to give informed written consent for both the circumcision and for participation in the study. To be eligible, infants had to be between 24 hours and 60 days old, have a weight-for-age greater than -2 z-scores against the WHO standards, no history of familial bleeding disorder, uncircumcised penis and genital anatomy of normal appearance, normal physical examination and general good health (e.g., no fever, rashes or jaundice). Every parent of a circumcised infant received an appointment card with the cell phone number for a 24-hour hotline, which was staffed on a 24-hour basis by an on-call trained clinician.

All providers were trained on correct management, recording and reporting of AEs. Emergency procedures were in place, including a parental hotline, a fully stocked emergency kit at each facility, a project vehicle and driver at the ready, a standby pediatric surgeon within a maximum two-hour drive. All emergency first-responders were trained in European pediatric life support. The system for ascertaining and treating AEs was consistent with the WHO “Manual for Early Infant Male Circumcision under Local Anaesthesia” [9]. All intra-operative and post-operative AEs and outcomes were recorded as mild, moderate or severe. Only moderate and severe AEs and deaths are reported here. The information collected included: type of AE, severity, name and cadre (DM, Clinical Officer, nurse) of the operator, how the AE was treated, follow-up recommendations and clinical notes. One project coordinator in each Division was assigned to review records weekly for quality assessment and improvement when needed. Moderate or severe AEs were reported immediately to the District Coordinators. Unusual, severe or recurring AEs were discussed in case review meetings and refresher training was provided, as needed.

Parents were asked to return to the facility 3–4 days post-operatively to have the infant’s wound assessed. Those not returning for review were contacted, usually by cell phone, and encouraged to come to a facility, or they were asked about the progress of the wound over the phone. The number and proportion of those coming for their post-operative review were recorded.

To evaluate uptake of EIMC, as well as demographic variables and levels of knowledge associated with uptake, survey questionnaires were administered to mothers coming to facilities with their baby son for the first Oral Polio Vaccine (OPV-1). Women aged 16 years and above were eligible for participation. Because 92% of infants receive the OPV-1 [21], a random sample of mothers bringing their babies for vaccination should be representative of all mothers delivering a baby boy in the region. Mothers were screened for study eligibility as they registered for vaccination services.

After undergoing informed written consent procedures, mothers were interviewed in a private location at the health facility in her language of choice (English, KiSwahili, or DhoLuo) by Research Assistants (RAs) fluent in all three languages using netbook computers. Mothers were asked for consent to contact the father of the baby for an interview and, if she agreed, provided locator information for the father. RAs traced the father and attempted to interview him in the location of his choice. All participants were given 200 KSH in compensation for their time. We used publicly available data from the Kenya Health Information System [22] to determine the number of OPV-1 vaccines administered at each facility in the calendar year prior to the survey. The number of surveys completed at each facility approximated the proportion of the division-level OPV-1 administered at that facility in the previous year.

### Statistical analyses

The main outcome variable of interest was whether the baby was circumcised or uncircumcised. This was assessed by inspection by the interviewer with approval from the mother. Independent variables of interest included whether in the SDP or SDPplus division, demographic variables, circumcision status of the father, and variables associated with being informed about EIMC, decision-making, and general endorsement of EIMC. We estimated bivariate and multivariate Poisson regression models with robust standard errors to compute prevalence ratios (PRs) and confidence intervals to assess differences between parents whose son was circumcised and those whose son was uncircumcised.

All variables significant at the p<0.05 level in bivariate regression were considered for inclusion in multivariate regression models. Variables associated with treatment group were investigated for potential confounding effects. Due to collinearity between mother and father responses (e.g. education, religion), we selected one or the other based on Wald tests. In addition, since not all fathers completed an interview, we created variables that supplemented the father’s information with information provided by the mother for this analysis (e.g., father’s circumcision status). We first estimated a model including treatment group and sociodemographic variables (Model 1). Next, we added variables associated with knowledge and decision-making (Model 2). Model fit was assessed by examining the Hosmer and Lemeshow Goodness-of-fit statistic from the final models. Unless otherwise specified, a predetermined significance level of p ≤0.05 was used to assess significance for all tests. Stata v13 [23] was used for analyses.

The Kenyatta National Hospital/University of Nairobi Ethics and Research Committee and the University of Illinois at Chicago Office for the Protection of Research Subjects provided ethical approval for this study.

## Results

### Early infant circumcisions performed

We do not have estimates of how many parents and guardians were informed about and offered EIMC for their babies, since mobilization and demand creation occurred throughout the community among the general population and in multiple venues in health facilities. A total of 2233 mothers consented to have their baby boy circumcised. One hundred thirteen babies (5.3%) were ineligible for circumcision: 2 were older than 60 days; 43 were low weight-for-age; 16 presented with fever; 16 had penile or scrotal abnormalities; 13 had jaundice; and 23 were excluded for other reasons (e.g. respiratory problems, rash, hypoglycemia, delayed micturition). Three mothers consented, but then did not have their baby circumcised. Therefore, the final sample of early infants circumcised was 2117.

### Characteristics of infants circumcised and by whom

**Fig 1:**
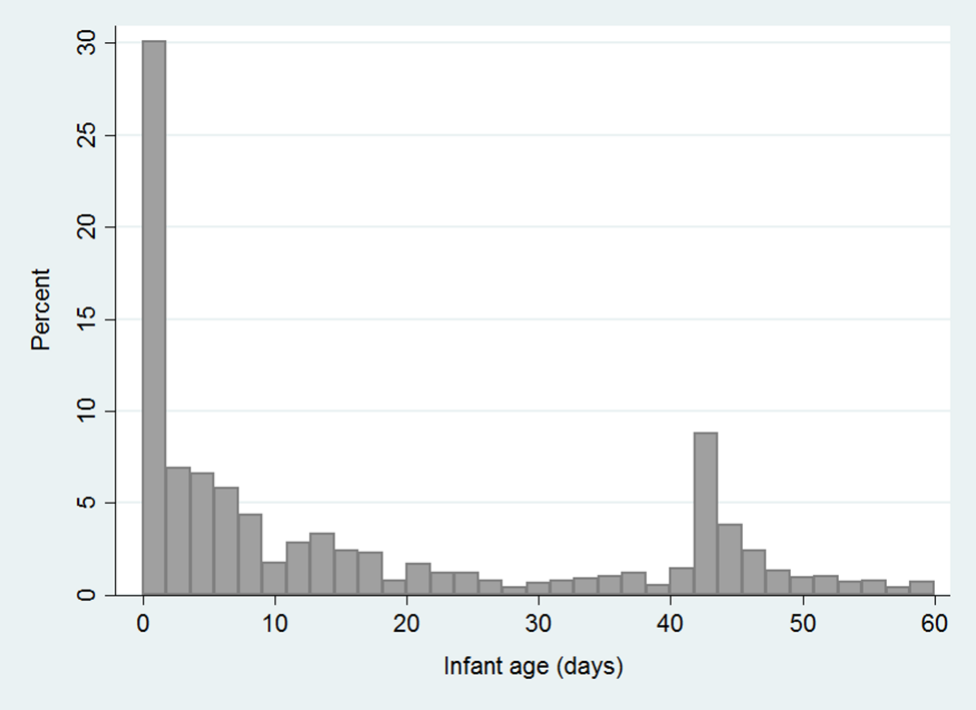
Age of infants at time of circumcision. Median age 8 days (IQR 1,36).

There were 766 infant boys circumcised in the SDP division and 1351 in the SDPplus division. The distribution of the infants by age in days is shown in Fig 1. The median age of the 2117 infants was 8 days (IQR 1, 36), and the median weight was 3.6 kg (IQR: 3.2, 4.4) with those in the SDPplus division older (median 10 days versus 4 days) and heavier (median 3.7kg versus 3.5kg) (Table 1). Overall, the largest age group were those circumcised at less than one week of age (46%), with 72.5% of all infants circumcised by 30 days of age. A secondary peak of circumcisions occurred around age 42 days. This is when mothers are instructed to bring their babies for OPV-1, thus presenting an opportunity to educate and recruit mothers who may not have been informed or able to make a decision earlier. More babies in the SDPplus division were circumcised at this later time than in the SDP division. The great majority of mothers (87.9%) in the SDP division reported mobilizers and CHWs to be their main source of EIMC information compared to 61.4% of mothers in the SDPplus division, where more mothers (37.5% versus 11.1%) cited maternity and the MCH clinic as their main sources of information. Overall, just 9.6% of mothers reported delivering outside a health facility–more in the SDPplus division (11%) compared to the SDP division (7.2%).

**Table 1:** Early infant male circumcisions performed by treatment group (N = 2117).

*Variable*	*Total*		SDP (n = 766)		SDPplus (n = 1,351)			
	*Median*	*Q1*, *Q3*	*Median*	*Q1*, *Q3*	*Median*	*Q1*, *Q3*	*Chisq*	p-value
**Infant age (days)**	8.0	(1, 36)	4.0	(1, 21)	10.0	(2, 41)	34.67	< 0.0001
**Weight (kg)** ^**a**^	3.6	(3.2, 4.4)	3.5	(3.1, 4.0)	3.7	(3.2, 4.5)	18.66	< 0.0001
**Time of EIMC procedure (min)** ^**b**^	12.0	(10, 14)	10.0	(10, 12)	13.0	(10, 15)	228.75	< 0.0001
	***n***	***%***	***n***	***%***	***n***	***%***		
**Infant age (days)**								
0–1	627	29.6	316	41.3	311	23.0	80.78	< 0.0001
2–10	537	25.4	166	21.7	371	27.5		
11–30	370	17.5	122	15.9	248	18.4		
31–60	583	27.5	162	21.1	421	31.2		
**Mother age (years)**								
15–19	211	10.0	76	9.9	135	10.0	9.71	0.046
20–24	496	23.4	162	21.1	334	24.7		
25–29	557	26.3	187	24.4	370	27.4		
30–34	570	26.9	226	29.5	344	25.5		
35+	283	13.4	115	15.0	168	12.4		
**Father age (years)**								
15–19	20	0.9	7	0.9	13	1.0	10.58	0.032
20–24	202	9.5	66	8.6	136	10.1		
25–29	408	19.3	143	18.7	265	19.6		
30–34	531	25.1	170	22.2	361	26.7		
35+	956	45.2	380	49.6	576	42.6		
**Main source of EIMC information**								
Mobilizer/CHW	1502	70.9	673	87.9	829	61.4	168.94	< 0.0001
Maternity/MCH	592	28.0	86	11.2	506	37.5		
Other	23	1.1	7	0.9	16	1.2		
**Site of delivery**								
Home	204	9.6	55	7.2	149	11.0	8.40	0.004
Facility	1910	90.2	711	92.8	1199	88.7		
**Circumcision service**								
Home	365	17.2	0	0.0	365	27.0	250.07	< 0.0001
Facility	1752	82.8	766	100.0	986	73.0		
**Designation**								
Direct Hire	1419	67.0	727	94.9	692	51.2	438.20	< 0.0001
Domiciliary Midwife	541	25.6	9	1.2	532	39.4		
MOH	157	7.4	30	3.9	127	9.4		
**Clinician Cadre**^**c**^								
Clinical officer	614	29.0	304	39.7	461	34.1	66.2	< 0.0001
Nurse	1498	70.8	310	40.5	1037	76.8		
**Client followed up**								
Yes	1563	73.8	594	77.5	969	71.7	8.57	0.003
No	554	26.2	172	22.5	382	28.3		

^a^ n = 2107; 1 missing in SDP, 9 missing in SDPplus

^b^ n = 2114; 3 missing in SDPplus

^c^ n = 2112, 1 missing in SDP, 4 missing in SDPplus

Regarding the EIMC procedure, nearly all took 15 minutes or less with those performed in the SDP division taking less time than those in the SDPplus division. In the SDPplus division, nearly 40% of procedures were performed by DMs and about half (51.2%) by Direct Hires ((i.e., those staff added to the facility by the project); whereas, in the SDP division nearly all (94.9%) were performed by the Direct Hire staff, with very few procedures (7.4%) performed by regular MOH staff in either division. As expected, all the circumcisions performed in homes were in the SDPplus division where the DMs operated, with 365 circumcisions representing 27% of all procedures perfomed in the SDPplus division. All mothers were urged to bring their babies back to the facility or to the DM 3–4 days after the procedure. Follow-up rates overall were 73.8%—higher in the SDP division (77.5%) than in the SDPplus division (71.7%) (p = 0.003)

### Adverse events

Out of 2117 circumcisions, there were 6 moderate AEs (0.28%); all were related to EIMC and all occurred post-operatively. Two were cases of the baby returning with bleeding from the wound, which required a suture, and four were infections which resolved with administration of an antibiotic (ampicillin/cloxacillin syrup). Three of the six cases of moderate AEs were in the SDPplus Division (AE rate 0.22%) and three were in the SDP Division (AE rate 0.39%). There were five severe AEs (0.24%), all of them entailing an injury to the glans penis and requiring hospitalization and corrective surgery by the consultant pediatric surgeon. Three were in the SDP division, one was in the SDPplus division and one was at the UNIM Research and Training Center in Kisumu and occurred in the context of training. Four of the five injuries to the glans occurred either during training (n = 1) or within two weeks after the provider had been trained and had started practicing (n = 3). These cases required emergency transport from the health facility where the injury occurred to a referral center 10 to 150 minutes away by road. The babies were hospitalized for two to seven days and were followed-up for weeks thereafter to ensure full function of the penis. The cases healed fully with pieces of tissue of varying sizes missing from the glans penis. Notably, there were no AEs, either moderate or severe, that occurred in the context of the 365 procedures performed outside health facilities.

There were 3 deaths, two unrelated to the surgery. One baby suffered a severe fracture of his skull in the home and the other unrelated case was a baby initially diagnosed with neonatal sepsis and later found to have a heart defect upon autopsy. The third death was one of the cases of injury to the glans. The baby was put under general anesthesia for corrective surgery, came out of anesthesia, seemed well-recovered three hours after surgery, was placed in a bed with his mother and was sleeping with his mother, but was discovered deceased when she awoke one hour later. This death was determined to be “probably related” to EIMC since it would most likely not have occurred had the baby not been circumcised, but the proximal cause of the death was not clearly established.

### Mother and father recruitment and sample size

Of 22678 mothers who came to the facilities with a baby, 1841 (8.1%) came with a baby boy for OPV-1 services and 1768 (96%) were approached by a RA. Seventy-three mothers (4.1%) declined to participate, most citing inadequate time for the interview; 16 mothers (1.0%) were below 16 years of age; and 1679 were interviewed. There was insufficient or ambiguous location information collected on 19 (1.1%) of these women. Therefore, a total of 1660 consented mothers are included in this analysis.

Of the 1679 mothers who agreed to be interviewed, 1501 (89.3%) gave approval to contact the father of the baby and 1288 (85.8%) of these fathers were contacted. Fourteen men refused to participate; 1274 (98.9%) consented and were interviewed, but 15 (1.2%) men could either not be matched in the dataset to a mother or there was ambiguous location information on the mother. Therefore, the final sample of fathers included in this analysis is 1259 men or 76% of fathers of babies whose mothers were interviewed.

#### Characteristics of the mothers and fathers interviewed

Table 2 shows characteristics of the mothers and fathers interviewed by treatment group. The mean age of the mother participants was 25.2 years and there was no difference between the groups. Fathers were on average 2 years older in the SDP division compared to the SDPplus division (33 vs. 31). Fathers in the SDPplus division were more likely to be unmarried, while marital status for mothers was similar across groups. The groups differed on education level of mothers and fathers, with more secondary education in SDPplus. Mothers and fathers in the SDPplus division were more likely to be Christian and less likely to be Muslim or other minority religion, while there were more minority ethnicity fathers in SDPplus (4% vs. 1%). More fathers were circumcised in the SDP division (44% vs. 38%), and mothers in the SDP division were more likely to report an HIV positive or unknown status (27% vs. 18%).

**Table 2:** Characteristics of mothers (*N* = 1,660^a^) and fathers (N = 1,259)^b^ in Rachuonyo, western Kenya, by treatment group, Mtoto Msafi Mbili Study, September 2014 –July 2016.

		Treatment group					
		SDP		SDPplus			
**Characteristics**	***n* total**	***n***	***mean (SD)***	*** *** ***n***	***mean (SD)***	***t***	***p***
Mother's age	1,660	799	25.1 (5.8)	861	25.3 (5.5)	-0.72	0.474
Father's age	1,258	561	33.4 (8.9)	697	31.3 (5.7)	4.98	< 0.001
	***n total***	***n***	***%***	***n***	***%***	***Chisq***	***p***
Mother's education ^c^							
None or Primary	904	491	61.5	413	48.0	25.46	< 0.001
Secondary	600	252	31.5	348	40.4		
Post-Secondary	156	56	7.0	100	11.6		
Father's education ^c^							
None or Primary	483	307	54.7	176	25.2	69.00	< 0.001
Secondary	561	172	30.7	389	55.7		
Post-Secondary	215	82	14.6	133	19.1		
Mother's marital status							
Married / live-in partner	1,363	663	83.0	700	81.3	0.79	0.37
Single, separated, widowed, or divorced	297	136	17.0	161	18.7		
Father's marital status							
Married / live-in partner	1,122	534	95.2	588	84.2	38.43	< 0.001
Single, separated, widowed, or divorced	137	27	4.8	110	15.8		
Mother's religion							
Christian	1,289	579	72.5	710	82.5	31.22	< 0.001
Muslim	33	27	3.4	6	0.7		
Other	338	193	24.2	145	16.8		
Father's religion							
Christian	1,020	440	78.4	580	83.1	14.15	0.001
Muslim	25	20	3.6	5	0.7		
Other	214	101	18.0	113	16.2		
Father's ethnicity							
Luo	1,224	554	98.8	670	96.0	8.79	0.003
Other	35	7	1.2	28	4.0		
Father's circumcision status							
Uncircumcised	744	314	56.0	430	61.6	3.94	0.047
Circumcised	514	246	43.9	268	38.4		
Mother's self-perceived HIV status							
HIV negative	1,285	583	73.0	702	81.5	35.22	< 0.001
HIV positive	287	148	18.5	139	16.1		
Not sure or refused to answer	88	68	8.5	20	2.3		

^a ^19 mothers were excluded due to un-classified treatment group (lived in neither Rachuonyo North nor South)

^b ^6 father surveys could not be matched to a mother survey, and 9 were excluded due to unclassified treatment group (mother lived in neither Rachuonyo North nor South)

^c^ Kruskal-Wallis test for trend reported

Table 3 shows mothers’ responses to questions concerning receipt of information about EIMC, and discussion with the father of the infant. In both divisions, over 90% of women received information; however, mothers in the SDP division were more likely to report that they received this information from a community health worker (12% vs. 7%), and before delivery (51% vs. 33%). Discussion with the father was similar between the groups, with 34% of mothers indicating that they did not discuss infant circumcision with the father.

**Table 3:** Information and discussion among mothers (*N* = 1,660^a^) in Rachuonyo, western Kenya, by treatment group, Mtoto Msafi Mbili Study, September 2014 –July 2016.

		Treatment group					
		SDP		SDPplus			
Characteristics	*n* total	*n*	%	* n*	%	*Chisq*	*p*
Received information about male infant circumcision							
No	149	77	9.6	72	8.4	0.82	0.364
Yes	1,511	722	90.4	789	91.6		
Received information from community health worker							
No	1,509	707	88.5	802	93.1	10.89	0.001
Yes	151	92	11.5	59	6.9		
Received information in hospital consultation							
No	464	236	29.5	228	26.5	1.92	0.166
Yes	1,196	563	70.5	633	73.5		
Received information ^b^							
Before pregnancy	143	96	12.0	47	5.5	51.17	< 0.001
During pregnancy	547	311	38.9	236	27.4		
At delivery	518	209	26.2	309	35.9		
After delivery	287	97	12.1	190	22.1		
Today or did not receive	165	86	10.8	79	9.2		
Discussed with father of child ^b^							
Before birth	73	37	4.6	36	4.2	2.02	0.156
At delivery	360	212	26.5	148	17.2		
After delivery	644	261	32.7	383	44.5		
Did not discuss	569	286	35.8	283	32.9		

^a ^19 participants were excluded due to un-classified treatment group (lived in neither Rachuonyo North nor South)

^b^ Kruskal-Wallis test for trend reported

### Circumcision uptake

#### Mothers

Overall, 425 mothers (25.6%) had babies who were circumcised (Table 4): 189/799 (23.7%) in the SDP division and 236/861 (27.4%) in the SDPplus division (chi-square = 3.07; p = 0.08). Mothers who resided in the SDPplus division were more likely to have their baby circumcised compared to those residing in the SDP division, but the difference was not statistically significant (PR = 1.16; 95% confidence interval [CI]: 0.98–1.37). Women who had a post-secondary education (PR = 1.54; 95% CI: 1.22–1.96), were unmarried and without a live-in partner (PR = 1.39; 95% CI: 1.16–1.66), or were Muslim (PR = 1.90; 95% CI: 1.32–2.73) were more likely to have circumcised their baby son. Mothers reported that 679 (41%) of the fathers were circumcised, 852 (51%) were uncircumcised, and 129 (8%) of mothers were unsure of the father’s circumcision status. When mothers reported the father to be circumcised, the son was significantly more likely to be circumcised (PR = 1.61; 95% CI: 1.36–1.91).

**Table 4:** Characteristics of mothers (*N* = 1,660^a^) in Rachuonyo, western Kenya, by circumcision status of their infant male, Mtoto Msafi Mbili Study, September 2014 –July 2016.

	Infant Male Circumcision							
	Circumcised		Not Circumcised		Unadjusted Prevalence Ratio			
Characteristics and Categories	*n*	%^b^	*n*	%^b^	PR	*95% Conf*. *Int*.		*p*
Treatment								
SDP	189	23.7	610	76.3	1.00			
SDPplus	236	27.4	625	72.6	1.16	(0.98,	1.37)	0.081
Age, *mean* (*SD*)	425	25.2 (5.9)	1,235	25.1 (5.6)	1.00	(0.99,	1.02)	0.917
Highest educational attainment								
None or Primary	214	23.7	690	76.3	1.00			
Secondary	154	25.7	446	74.3	1.08	(0.91,	1.30)	0.377
Post-Secondary	57	36.5	99	63.5	1.54	(1.22,	1.96)	< 0.001
Marital status								
Married or with live-in partner	326	23.9	1,037	76.1	1.00			
Single, separated, widowed, or divorced	99	33.3	198	66.7	1.39	(1.16,	1.68)	< 0.001
Religion								
Christian	329	25.5	960	74.5	1.00			
Muslim	16	48.5	17	51.5	1.90	(1.32,	2.73)	0.001
Other	80	23.7	258	76.3	0.93	(0.75,	1.15)	0.488
Infant’s father ethnicity								
Luo	414	25.8	1,188	74.2	1.00			
Other	11	19	47	81	0.73	(0.43,	1.26)	0.26
Infant’s father circumcision status								
Uncircumcised	172	20.2	680	79.8	1.00			
Circumcised	221	32.5	458	67.5	1.61	(1.36,	1.91)	< 0.001
Not sure	32	24.8	97	75.2	1.23	(0.88,	1.71)	0.220
Self-perceived HIV status								
HIV negative	319	24.8	966	75.2	1.00			
HIV positive	90	31.4	197	68.6	1.26	(1.04,	1.54)	0.019
Not sure or refused to answer	16	18.2	72	81.8	0.73	(0.47,	1.15)	0.178
Received information about male infant circumcision								
No	4	2.7	145	97.3	1.00			
Yes	421	27.9	1,090	72.1	10.38	(3.93,	27.39)	< 0.001
Received information from community health worker								
No	109	25.4	1,126	74.6	1.00			
Yes	42	27.8	109	72.2	1.10	(0.84,	1.44)	0.508
Received information in hospital consultation								
No	68	14.7	396	85.3	1.00			
Yes	357	29.9	839	70.2	2.04	(1.61,	2.58)	< 0.001
Received information								
Today or did not receive	5	3.0	160	97	1.00			
Before pregnancy	51	35.7	92	64.3	11.77	(4.83,	28.69)	<0.001
During pregnancy	159	29.1	388	70.9	9.59	(4.01,	22.97)	<0.001
At delivery	145	28.0	373	72	9.24	(3.85,	22.15)	<0.001
After delivery	65	22.7	222	77.4	7.47	(3.07,	18.19)	<0.001
Discussed with father of child								
Did not discuss	71	12.5	498	87.5	1.00			
Before birth	34	46.6	39	53.4	3.73	(2.69,	5.18)	<0.001
At delivery	112	31.1	248	68.9	2.49	(1.91,	3.25)	<0.001
After delivery	205	31.8	439	68.1	2.55	(2.00,	3.26)	<0.001

PR = prevalence ratio.

^a ^19 participants were excluded due to un-classified treatment group (lived in neither Rachuonyo North nor South)

^b ^Mean (SD) for continuous variable.

Circumcision of the infant was associated with mothers’ beliefs about their own likelihood of HIV infection, with the information that they had received about circumcision, and with whom they had consulted about the decision. Those mothers who perceived themselves to be HIV positive were more likely to have their son circumcised (PR = 1.26; 95% CI: 1.04–1.54). If the mother had received information about EIMC, she was over ten times more likely to have her son circumcised than if she had not received information (PR = 10.4; 95% CI: 3.93–27.4). If she received the information from a CHW, she was no more likely to have the baby circumcised (PR = 1.10; 95% CI: 0.84–1.44) than if she had received no information, but if information came during a hospital consultation (PR = 2.04; 95% CI = 1.61–2.58), the baby was more likely to be circumcised. There was a greater effect when information was received earlier–before pregnancy compared to after delivery–and information received during pregnancy or delivery had intermediate effects. Very importantly, discussing EIMC with the father of the baby influenced EIMC uptake significantly. The prevalence of the baby being circumcised—whether the discussion occurred before birth, around time of delivery or after delivery—at least two and a half times greater when the father was consulted than when he was not. The effect was greater when discussion occurred before birth (PR = 3.73) vs. at delivery (PR = 2.49) or after delivery (PR = 2.55). Mothers of circumcised babies believed their son was less likely to be “somewhat” (PR = 0.39; 95% CI: 0.31–0.49) or “very much” (PR = 0.14; 95% CI: 0.07–0.29) at risk of acquiring HIV.

#### Fathers

Of the 1259 fathers who were contacted, consented and interviewed, 333 (26.5%) were fathers of babies who were circumcised and 925 (73.5%) were fathers of babies who were uncircumcised at the time that the mother was interviewed at the health facility (Table 5). The fathers in both groups were the same mean age (32 years) and there was no significant difference in the prevalence of babies circumcised in the SDP division versus the SDPplus division (PR = 1.19; 95% CI: 0.99–1.44). Fathers who had a secondary (PR = 1.24; 95% CI: 1.00–1.53) or a post-secondary education (PR = 1.46; 95% CI: 1.13–1.88) were more likely to have a circumcised infant son than those with a primary school education or less. Those fathers who were Muslim (PR = 2.16; 95% CI: 1.51–3.11), and who had received information about EIMC (PR = 2.26; 95% CI: 1.50–3.40) were more likely to have a circumcised baby. Consistent with the reports from the mothers, the proportion of men who reported that they were circumcised was 41%, and there was greater prevalence of circumcision among their sons than among the sons of men who were not circumcised (PR = 1.57; 95% CI: 1.31–1.89). Similar to reports from the mothers, fathers who discussed EIMC with the mother of the infant were more likely to have a circumcised baby, especially when the discussion occurred before birth (PR = 4.44; 95% CI 2.80–7.03), but also when discussion occurred at delivery (PR = 2.89; 95% CI 1.92–4.33) or after delivery (PR = 2.62; 95% CI 1.79–3.84). There were no differences between men whose sons were circumcised versus those whose sons were not circumcised with respect to marital status, ethnicity, their own perceived HIV status, or the perceived HIV status of the mother. As with the mothers, fathers of sons who were circumcised were much less likely to believe that their son is at risk of HIV infection.

**Table 5:** Characteristics of fathers (*N* = 1,259^a^) in Rachuonyo, western Kenya, and circumcision status of male infant at OPV-1 visit, Mtoto Msafi Mbili Study, September 2014 –July 2016.

		Infant Male Circumcision							
		Circumcised		Not Circumcised		Unadjusted Prevalence Ratio			
Characteristics and Categories	*n* total	*n*	%^b^	*n*	%^b^	PR	*95% Conf*. *Int*.		*p*
Infant’s Mother Treatment Group									
SDP	561	134	23.9	427	76.1	1.00			
SDPplus	698	199	28.5	499	71.5	1.19	(0.99,	1.44)	0.066
Age^c^, *mean* (*SD*)	1,258	333	32.4 (7.7)	925	32.2 (7.2)	1.00	(0.99,	1.02)	0.624
Highest educational attainment									
None or Primary	483	108	22.4	375	77.6	1.00			
Secondary	561	155	27.6	406	72.4	1.24	(1.00,	1.53)	0.052
Post-Secondary	215	70	32.6	145	67.4	1.46	(1.13,	1.88)	0.004
Marital Status									
Married or with live-in partner	1,122	294	26.2	828	73.8	1.00			
Single, separated, widowed, or divorced	137	39	28.5	98	71.5	1.09	(0.82,	1.44)	0.566
Religion									
Christian	1,020	264	25.9	756	74.1	1.00			
Muslim	25	14	56	11	44	2.16	(1.51,	3.11)	< 0.001
Other	214	55	25.7	159	74.3	0.99	(0.77,	1.28)	0.956
Ethnicity									
Luo	1,224	325	26.6	899	73.5	1.00			
Other	35	8	22.9	27	77.1	0.86	(0.46,	1.59)	0.633
Self-circumcision status									
Uncircumcised	744	159	21.4	585	78.6	1.00			
Circumcised	514	173	33.7	341	66.3	1.57	(1.31,	1.89)	< 0.001
Self-perceived HIV status									
HIV negative	990	264	26.7	726	73.3	1.00			
HIV positive	141	39	27.7	102	72.3	1.04	(0.78,	1.38)	0.802
Not sure or refused to answer	128	30	23.4	98	76.6	0.88	(0.63,	1.22)	0.443
Perceived HIV status of mother									
HIV negative	982	254	25.9	728	74.1	1.00			
HIV positive	126	39	31.0	87	69.1	1.20	(0.90,	1.59)	0.211
Not sure or refused to answer	151	40	26.5	111	73.5	1.02	(0.77,	1.36)	0.87
Received information about infant male circumcision									
No	166	21	12.7	145	87.4	1.00			
Yes	1,093	312	28.6	781	71.5	2.26	(1.50,	3.40)	< 0.001
Received information									
Today or did not receive	170	22	12.9	148	87.1	1.00			
Before pregnancy	152	42	27.6	110	72.4	2.14	(1.34,	3.41)	0.001
During pregnancy	198	50	25.3	148	74.7	1.95	(1.23,	3.08)	0.004
At delivery	463	139	30.0	324	70.0	2.32	(1.53,	3.51)	< 0.001
After delivery	276	80	29.0	196	71.0	2.24	(1.45,	3.45)	< 0.001
Discussed with mother of child									
Did not discuss	240	26	10.8	214	89.2	1.00			
Before birth	52	25	48.1	27	51.9	4.44	(2.80,	7.03)	< 0.001
At delivery	259	81	31.3	178	68.7	2.89	(1.92,	4.33)	< 0.001
After delivery	708	201	28.4	507	71.6	2.62	(1.79,	3.84)	< 0.001

PR = prevalence ratio.

^a ^Six father surveys could not be matched to a mother survey, and nine participants were excluded due to unclassified treatment group (mother lived in neither Rachuonyo North nor South)

^b ^Mean (SD) for continuous variable

^c ^One missing value.

### Multivariable modeling

The predictors of uptake of EIMC included in the first multivariable model in addition to treatment group were mother’s education level, her marital status, her self-perceived HIV status, and father’s ethnicity, religion, and circumcision status (Table 6). Adjusting for these potential confounders, the prevalence of EIMC was greater among parents who resided in the SDPplus division than among those who live in the SDP division (aPR = 1.22, 95% CI: 1.03–1.45). All covariates also had statistically significant effects.

**Table 6:** Multivariate models of factors associated with infant male circumcision in Rachuonyo, western Kenya, Mtoto Msafi Mbili Study, September 2014 –July 2016, N = 1660.

	Model 1				Model 2			
	Treatment Group and Demographics				Treatment Group, Demographics, and Information and Discussion			
Variable and Categories	aPR	95% Conf Int.		p	aPR	95% Conf Int.		p
Infant’s Mother Treatment Group								
SDP (ref.)	1.00				1.00			
SDPplus	1.22	1.03	1.45	0.024	1.23	1.04	1.45	0.016
Father's ethnicity								
Other	1.00				1.00			
Luo	1.73	1.00	2.97	0.049	1.40	0.82	2.37	0.217
Mother’s Education								
None or Primary (ref.)	1.00				1.00			
Secondary	1.01	0.84	1.22	0.889	0.96	0.81	1.14	0.637
Post-Secondary	1.50	1.17	1.93	0.002	1.33	1.04	1.69	0.021
Mother’s Marital Status								
Married or with live-in partner (ref.)	1.00				1.00			
Single, separated, widowed, or divorced	1.33	1.08	1.62	0.006	5.67	3.31	9.69	< 0.001 ^b^
Religion^a^								
Christian (ref.)	1.00				1.00			
Muslim	2.18	1.55	3.06	< 0.001	1.85	1.29	2.65	0.001
Other	1.10	0.88	1.37	0.394	1.14	0.92	1.40	0.222
Father’s Circumcision Status^a^							
Uncircumcised (ref.)	1.00				1.00			
Circumcised	1.47	1.23	1.77	< 0.001	1.34	1.13	1.59	0.001
Unknown	1.17	0.71	1.91	0.534	1.17	0.71	1.93	0.548
Mother’s Self-Perceived HIV Status								
HIV negative (ref.)	1.00				1.00			
HIV positive	1.41	1.16	1.72	0.001	1.39	1.15	1.67	0.001
Unknown	0.80	0.51	1.27	0.349	0.88	0.56	1.39	0.594
Mother: Received information								
No information before today					1.00			
Before pregnancy					5.72	2.71	3.68	< 0.001
During pregnancy					4.81	2.21	3.42	0.001
At delivery					4.59	2.12	3.30	0.001
After delivery					3.79	1.77	2.85	0.004
Mother: Received hospital consultation								
No					1.00			
Yes					1.33	1.06	1.67	0.014
Mother: Discussed with father of child								
No					1.00			
Yes					5.39	3.31	8.80	< 0.001^c^
Mother's marital status x Discussed with father of child								
Unmarried x Yes					0.23	0.13	0.41	< 0.001

^a^ Father’s self-reported information if available, otherwise reported by mother

^b^ Effect of marital status when no discussion with father of child

^c^ Effect of discussion when mother's marital status is married or living with partner

In the second model we added variables related to levels and sources of information about EIMC, including the timing of mother receiving information about EIMC, her receiving information during a hospital consultation, and discussion of EIMC with the father. We also included the interaction effect of discussion with the father and marital status. Adjusting for these variables did not change the effect of treatment group; (aPR = 1.23, 95% CI 1.04–1.45). The strongest effects were the mother having received information about EIMC at any time (Chi2(4) = 18.23, p = .001), with earlier information having larger effects, and the mother having discussed circumcision with the father if they were married or cohabiting; (aPR = 5.39, 95% CI 3.31–8.80),. Most unmarried women (67%) did not discuss infant circumcision with the father, and discussion of EIMC with the father among single mothers had no significant effect on prevalence of circumcision of the baby.

## Discussion

To address the lack of evidence regarding introduction of EIMC services in sub-Saharan African settings, we conducted a simultaneous, prospective comparison of two models of EIMC service delivery: a standard delivery package (SDP) that included health facility-based provision of EIMC services with community engagement for client referral versus a standard package plus (SDPplus) that included community-delivered IMC services. We found that uptake of EIMC by parents for baby boys up to 60 days of age was 25.6% with a moderate increase in uptake in the SDPplus division. Babies were more likely to be circumcised if the mother had greater than a secondary education, if she knew or perceived herself to be HIV-positive, if she were single, if the father was circumcised and if the father was a Muslim. Also important was the mother’s and father’s exposure to information. Circumcision of the baby was much more likely if the mother had received information about EIMC and if the father and mother had discussed the EIMC decision together.

The study was designed to test the difference between a primarily facility-based model versus a facility plus community-based model of integrating EIMC with MNCH services because in a previous study we found that approximately 45% of mothers do not deliver at health facilities and that fathers’ involvement has a significant impact on the EIMC decision [16]. We expected significantly greater uptake in the SDPplus model since the domiciliary midwives, by living in the community, could identify pregnant women and engage both the woman and the father of the child early, before or very soon after delivery. We did find that mothers who received information about EIMC before pregnancy and discussed the decision with the father before delivery were more likely to have their baby circumcised; however, this did not result in a large difference in circumcision uptake between the two models. Some of the challenges of the SDPplus model were the retention of staff; nurses and clinical officers would prefer to be stationed at a facility rather than cover large areas on foot or with a bicycle or motorbike, all of which means were available to them. As soon as a position opened up at any facility in the area, they resigned from their position and moved to where a more anchored position was available. Therefore, there was very high turnover of the DMs, and new persons had to be recruited and trained who were not as familiar with or as well accepted in the communities where they served. Of the four original DMs trained and hired, just one remained in the position throughout the two years of the project. In total, over the course of the project, we recruited, trained and hired 8 DMs for the four positions; some positions remained vacant for varying periods of two to five months as we recruited and trained replacements. High staff turnover is likely to be a challenge for many EIMC programs [24]. Unless domiciliary midwives are given inducements to remain at their stations for substantial periods, the SDPplus model is unlikely to add significantly to EIMC uptake, and it is also unlikely to be cost effective relative to adult and adolescent VMMC [18]. Another reason the SDPplus model may not have resulted in significantly greater EIMC uptake was the 2013 introduction of free MNCH services at all Kenyan government health facilities [25]. This initiative seems to have been successful as far as encouraging women to deliver at health facilities rather than at home. In our sample, just 5.3% of mothers reported having delivered outside a health facility.

This study aimed to attain a level of 30% uptake of EIMC. Our weighted random sample of mothers coming to facilities with their babies for OPV-1 showed that uptake was approximately 26% in the catchment area. While lower than our original 30% target, it nevertheless represents a significant achievement given 0 babies were found to be circumcised at baseline [19], and approximately 15% uptake toward the end of our previous pilot study [16]. The Kenyan Government target for EIMC uptake is: “To circumcise at least 40% of male infants who come into contact with EIMC-providing facilities within 60 days after birth by 2019.” [6] This may be difficult to achieve. For example, attempts to scale up EIMC in other sub-Saharan African countries have had even lower success than our study in Kenya. A pilot project in Tanzania lasting 19 months attained a circumcision prevalence of 16.4% of all male infants born in 8 health facilities [26]. In Zimbabwe, in a study of the AccuCirc device, 11% uptake was attained [27], and in Zambia, despite 80% of mothers saying they would have their baby boy circumcised, uptake reached only 11% [28]. A study in Lesotho attempting to scale up EIMC also achieved 11% uptake [29]. Swaziland has achieved possibly the highest number of EIMCs in east and southern Africa– 5149 EIMCs performed between 2010 and 2014 –however, without a denominator, it is not possible to calculate uptake [24]. Overall, program implementers and donors should be cautioned that, despite high uptake predicted by hypothetical acceptability studies [27,30], it is likely to take several years and allocation of substantial resources before rates of EIMC uptake may achieve targets of 40% or higher.

The significant effect of knowledge on acceptability of EIMC has been documented in other studies of EIMC uptake [27,31]. In this study, the factors most strongly associated with uptake of EIMC were related to whether mothers and fathers received information about the availability of and reasons for EIMC, and whether fathers were involved in the EIMC decision. In adjusted analyses, a mother was nearly five times more likely to have her son circumcised if she had received information about the risks and benefits of EIMC. In the bivariate results, receiving the information before pregnancy had a larger effect than being informed after delivery, with during pregnancy and at delivery having intermediate effects. This indicates that, despite 95% of mothers interviewed having delivered in a facility, informational and education campaigns (IEC) in the general community (e.g., radio spots, talks at markets, chiefs’ meetings, churches and social gatherings) may have the strongest influence on EIMC uptake, although information at ante-natal care (ANC) and in maternity will also improve uptake far greater than waiting until the OPV-1 visit before the mother is exposed to EIMC messages. Expansion of community education and awareness-raising for EIMC via media outlets, with special messages for fathers, has been advocated by others, along with encouragement for women coming to ANC to discuss EIMC with their partners, as means to greater EIMC acceptability [26,32].

Both the mother and father are clearly important in the EIMC decision. In our baseline survey for this study, three quarters of women and men felt that the EIMC decision should be made equally between parents [19], and our previous study in western Kenya showed only 13% of mothers who elected for their son to be circumcised did not consult the father [16]. Similarly, in Botswana, just 10% of mothers stated they would be willing to make the decision about infant circumcision by themselves [30], and fathers have been found to be crucial in the EIMC decision in studies from other sub-Saharan African countries [26,31]. These findings are confirmed by our results showing that married and co-habiting mothers who discussed the EIMC decision with the father of the child were approximately three times more likely to have their son circumcised. Thus, involving both mothers and fathers in EIMC programming will be important for successful implementation of services. Providing the time and means (e.g., phone credit) for mothers to consult with the father is likely to improve uptake significantly.

One of the often cited advantages of EIMC over adult VMMC its greater safety [6,9,33,34]. AEs in this study were few. Combining moderate and severe AEs, there was an AE rate of just 0.5%, which is lower or comparable to adult VMMCs done in similar settings [1–3,35] and well under the 2% maximum rate targeted by the Kenyan MOH [35]. However, while there were few, several of the AEs that did occur were serious and resulted in permanent adverse sequelae. When placing the Mogen clamp, the glans penis of five babies were injured and all of these cases required hospitalization and corrective surgery by a qualified pediatric surgeon. This points to the need for EIMC programs to have emergency procedures in place with trained surgeons ready to receive and address serious cases [9,35]. Two of the moderate cases required one or two stitches to staunch bleeding, requiring a clinician onsite who is capable of suturing. During the course of the study, we found that cases of injured glans could be minimized by ensuring that all foreskin adhesions are fully separated from the penile shaft and glans, thus minimizing risk of the glans being carried with the foreskin before clamping. In addition, four of the five injuries to the glans occurred when the provider was either in training or was in his or her first two weeks of practice. From these cases, we learned that providers need supervision beyond the 10 procedures necessary for certification to include the first 20–40 procedures after placement in a facility. Regular quality assessments by trainers as well as bringing providers together to share case reviews should also be part of a quality EIMC program. Currently, the Kenyan government has approved use of only the Mogen for its national EIMC program [36] and this seems to be the preferred device in use in many African EIMC programs. Nevertheless, the risk of injuring the glans remains when using the Mogen clamp [35,37–39]. Alternative devices, like the AccuCirc and Gomco clamp, which prevent risk of injury to the glans, should be considered. In a recent study by our group, providers who had experience using both the Mogen and AccuCirc, expressed the view that the AccuCirc would be safest and more appropriate for a national EIMC program [40].

The ages of babies circumcised in this study were restricted to between 24 hours to 60 days after birth, consistent with Kenya MOH guidelines. We are aware of one other EIMC program that extends the age window to 78 days [26], resulting in a median age of those circumcised of 22 days compared to 8 days in this study. This is in contrast to the program in Swaziland in which 80% of EIMCs took place in the immediate post-partum period before discharge. The majority of babies (55%) in our study were 10 days old or younger, and 73% were 30 days old or younger. Most babies recruited after 30 days were recruited when they were brought to a facility for their OPV-1 immunization around 42 days after birth. Since 92% of babies in the region get their OPV-1 vaccination, this is an opportune time to offer EIMC to those who were not circumcised in the first 30 days after birth. If the age of circumcision were restricted to only babies up to age 30 days, approximately 27% of boys might be missed, unless widespread education and information dissemination could increase the proportion of parents accepting EIMC earlier.

### Limitations

Our study has several limitations. It was conducted in only one county in Kenya. The county is primarily rural with just two large towns. Our results may not be generalizable to other parts of Kenya or to other countries in East and southern Africa where introduction of EIMC is being considered for sustained coverage of male circumcision for HIV prevention. The providers who participated in this study were all either clinical officers or nurses with back-up support from Medical Officers and a surgeon. Other countries may not have the cadre of Clinical Officer, and may not permit nurses to perform procedures. This could result in different outcomes, although several studies have shown equivalent safety profiles of EIMC performed by nurses and doctors [41]. We were able to contact and interview 76% of fathers. We do not know if this could have resulted in biased estimates of paternal attitudes either in favor or against EIMC.

Strengths of our study include: large sample size, enrolling both mothers and fathers of infant males, a sampling strategy designed to be representative of the general population of parents in the study area and an innovative attempt to integrate EIMC information and services with both facility and community-based MNCH services.

## Conclusions

The addition of community-based domiciliary midwives to a facility based model of integrating EIMC services with MNCH services was safe, but resulted in only moderate increases in uptake of EIMC and is unlikely to be cost effective. Information and education campaigns will be crucial to improving uptake of EIMC and must be extended outside health facilities to reach members of the general population, especially men and fathers, who are crucial to the EIMC decision. AEs are rare, but when they occur they can be serious and require efficient, reliable emergency back-up. Achieving a goal of 40% of baby boys being circumcised before age 60 days will face many challenges.

Kenya has a well-developed adult and adolescent VMMC program which is approaching saturation [6,32]. Beliefs and attitudes about EIMC will likely evolve as greater proportions of circumcised men enter into marriage and fatherhood. Our results should be of assistance to other sub -Saharan African countries considering scale-up of EIMC services as their adult VMMC programs mature.

## Supporting information

S1 File Kenya ministry of health early infant medical circumcision form. (PDF)

S2 File Mtoto Msafi Mbili Study Mothers’ questionnaire. (PDF)

S3 File Mtoto Msafi Mbili Study Fathers’ questionnaire. (PDF)
