# Supplementary material for: Prospective comparison of two models of integrating early infant male circumcision with maternal child health services in Kenya: The Mtoto Msafi Mbili Study
Source: PLoS One. 2017 Sep 7;12(9):e0184170. doi: 10.1371/journal.pone.0184170 (PMC5589171; doi:10.1371/journal.pone.0184170)
Supplement: S1 File — (PDF) [file pone.0184170.s001.pdf]

F: POST-OP FOLLOW-UP
(for any moderate or severe AE, inform appropriate supervisor and

F1: Date ☐☐ ☐☐ ☐☐☐☐
d d m m y y y y

F2: Type of visit ☐ 48hr ☐ 3/4 day ☐ 7 day ☐ Other specify________________________

F3: Post-op Adverse Event? ☐ No ☐ Yes
If Yes, refer to AE description form and complete the following

F4: Type of AE __________ F5: If Yes, how would you rate it? ☐ Mild ☐ Moderate ☐ Severe

F6: Notes:____

F7: Name of reviewing officer____________________________ Signature________________

F8: Date ☐☐ ☐☐ ☐☐☐☐
d d m m y y y y

F9: Type of visit ☐ 48hr ☐ 3/4 day ☐ 7 day ☐ Other specify________________________

F10: Post-op Adverse Event? ☐ No ☐ Yes
If Yes, refer to AE description form and complete the following

F11: Type of AE ____

F12: If Yes, how would you rate it? ☐ Mild ☐ Moderate ☐ Severe

F13: Notes:____

F14: Name of reviewing officer____________________________ Signature________________

G: Clinical Notes

____
____
____
____
____
____
____

Version 2 June 19, 2012

Ministry of Health
Infant Male Circumcision Client Form

Client Number: ☐☐☐☐☐-☐☐ Date ☐☐ ☐☐ ☐☐☐☐
d d m m y y y y

Theatre Register Number: (As per facility procedures) ☐☐☐☐☐

FACILITY/SITE DETAILS

Facility/Site Name ____

Service Delivery Type: Home ☐ Facility ☐ Other ☐ ______________

Village/Estate__________________________ Sub Location______________________

District__________________________ County______________________________

A: Client/Parent Information

A1: Infant Name ________________________ A2:DOB ☐☐ ☐☐ ☐☐☐☐
d d m m y y y y

A3: Mother's Name ____
First Name Middle Name Last Name

A4: Age ____________ A5: Telephone Contact ______________________________

A6: Father's Name ____
First Name Middle Name Last Name

A7: Age ____________ A8: Telephone Contact ______________________________

A9: Residence (infant): Village/Estate ________________ Sub Location ________________
District _________________________ County ______________________________

A10: Main source of VMMC information

☐ Radio/TV ☐ Print Media ☐ Road show ☐ Mobilizer/CHW ☐ Maternity/MCH

☐ Other (specify) ____

B: MEDICAL HISTORY AND PHYSICAL EXAMINATION
(Note: Check one box for each item)

B1: Family history of bleeding disorder ☐ Yes ☐ No

B2: Client presenting with

(1) Fever ☐ Yes ☐ No (2) Jaundice ☐ Yes ☐ No

(3) Undescended testis(es) ☐ Yes ☐ No (4) Scrotal Swelling ☐ Yes ☐ No

(5) Abnormal penile appearance ☐ Yes ☐ No

(6) Congenital Anomaly, (specify) ______________________________

(7) Other, (specify) ______________________________

B3: Pregnancy/Delivery

(1) Site of delivery ☐ Home ☐ Facility ☐ Other (2) Mode of delivery ☐ C-section ☐ Vag

(3) Pregnancy complications, (specify) ______________________________

B4: Any known allergies? ☐ Yes ☐ No If Yes, specify ______________________

B5: Weight: _________ kg

C: ELIGIBILITY FOR CIRCUMCISION

C1: Client eligible for circumcision?

C1.1: In good health ☐ Yes ☐ No

C1.2: Parents counseled ☐ Yes ☐ No

C1.3: Parent/guardian consent obtained ☐ Yes ☐ No

If No **for any of the above, explain** ______________________________

NOTE: PROCEED WITH CIRCUMCISION **ONLY** IF ALL BOXES HAVE BEEN CHECKED "YES"

D: CIRCUMCISION PROCEDURE

D1: Date of circumcision ☐☐ ☐☐ ☐☐☐☐
d d m m y y y y

D2: Start Time [24 hour clock]: |______|______| : |______|______|

D3: End Time |______|______| : |______|______|

D4: List pre operative medication given (if any)______________________________

D5: Anaesthesia used:

D5.1: Type	D5.2: Concentration	D5.3: Volume (mls)
☐ Lignocaine	☐ 1% ☐ 2%	____________
☐ Other (specify_____________)	____________	____________

D6: Type of circumcision procedure ☐ Mogen ☐ Other, (specify): ______________

D7: Adverse Events during operation? ☐ Yes ☐ No

D8: Type of AE ______________________________(refer to AE description form)

D9: If Yes, how would you rate it? (refer to AE description form) ☐ Mild ☐ Moderate ☐ Severe

D10: Name of clinician ______________________________

D11: Cadre: ☐ MO ☐ CO ☐ Nurse ______________________________
Signature

D12: Name of Assistant______________________________

D13: Cadre: ☐ MO ☐ CO ☐ Nurse ______________________________
Signature

E: IMMEDIATE POST-OP

E2: Medication given: ☐ Pannadol ☐ Other ______________________ ☐ None

E3: Parent/guardian given post-op instructions? ☐ Yes ☐ No

NOTE: IF A MODERATE OR SEVERE AE IS OBSERVED, INFORM APPROPRIATE SUPERVISER AND COMPLETE INCIDENCE FORM IMMEDIATELY
