# Supplementary material for: Prospective comparison of two models of integrating early infant male circumcision with maternal child health services in Kenya: The Mtoto Msafi Mbili Study
Source: PLoS One. 2017 Sep 7;12(9):e0184170. doi: 10.1371/journal.pone.0184170 (PMC5589171; doi:10.1371/journal.pone.0184170)
Supplement: S2 File — (PDF) [file pone.0184170.s002.pdf]

MTOTO MSAFI MBILI	Questionnaire - Mothers
**Date (dd/mm/yy): ____/____/____**	**Participant Number: ________________________**<br>**Site ID:_________**

[To be answered by the interviewer:]

1. *Start Time*

:

2. *Record geographic coordinates of location*

o	'	"	S

o	'	"	E

3. *Location of interview*
   ☐1 = Home of baby
   ☐2 = Home of respondent (but not baby)
   ☐3 = Health facility (specify): ________________________________
   ☐4 = Other (specify): ________________________________

Part 1: Demographic Information
[Read] I am going to start by asking you some questions about yourself, the baby and the father of the baby. Are you ready to begin?

4. What is your date of birth? *[dd/mm/yy]* ______/______/______

5. How old are you now? ___________ years old

6. What is your son's date of birth? *[dd/mm/yy]* ______/______/______

   [To be answered by the interviewer:]
   a. *Son receiving OPV I is:*
      ☐1 = ≤ 2 months old
      ☐0 = > 2 months old

7. What is your Ethnic origin? *[check one]*
   ☐ 1 = Luo
   ☐ 0 = Other (specify): ________________________________

8. What is the Ethnic origin of the father of the baby? *[check one]*
   ☐ 1 = Luo
   ☐ 0 = Other (specify): ________________________________

☐ 2 = Not sure

9. What is the circumcision status of the father of the baby? *[check one]*
   ☐ 1 = Circumcised
   ☐ 0 = Uncircumcised
   ☐ 2 = Not sure

   a. If **CIRCUMCISED**, when in his life was the father of the baby circumcised? *[check one]*
      ☐ 1 = Birth to eight weeks
      ☐ 2 = >8 weeks to <1 year old
      ☐ 3 = 1 to 9 years old
      ☐ 4 = 10 to 17 years old
      ☐ 5 = 18 to 29 years old
      ☐ 6 = 30 years or older
      ☐ 7 = Not sure

   b. If **CIRCUMCISED**, who circumcised him? *[check one]*
      ☐ 1 = A clinician
      ☐ 2 = A traditional circumciser
      ☐ 3 = Not sure
      ☐ 4 = Other (specify): ________________________________

10. What district do you currently reside in (spend the most nights sleeping in)? *[check one]*
    ☐ 1 = Rachuonyo North
    ☐ 2 = Rachuonyo South
    ☐ 3 = Other (specify): ________________________________

11. What is the highest level of school you completed? *[check one]*
    ☐ 0 = No level completed

    ☐ 1 = Class 1
    ☐ 2 = Class 2
    ☐ 3 = Class 3
    ☐ 4 = Class 4
    ☐ 5 = Class 5
    ☐ 6 = Class 6
    ☐ 7 = Class 7
    ☐ 8 = Class 8

    ☐ 9 = Form 1
    ☐ 10 = Form 2
    ☐ 11 = Form 3
    ☐ 12 = Form 4

    ☐ 13 = Post-graduate Certificate
    ☐ 14 = Post-graduate Diploma
    ☐ 15 = Post-graduate Degree

12. Are you currently employed? *[check one]*
    ☐ 1 = Yes
    ☐ 0 = No

13. What is your primary occupation? *[check one]*

☐ 1 = Hawker/Small Business owner
☐ 2 = Farmer
☐ 3 = Professional/Managerial
☐ 4 = Student
☐ 5 = Other (specify):_____________________________________

14. What is your current marital status? *[check one]*
☐ 1 = Not legally married, without a regular live-in partner
☐ 2 = Not legally married, with a regular live-in partner
☐ 3 = Legally married, not living with husband
☐ 4 = Legally married, living with husband
☐ 5 = Separated
☐ 6 = Widowed
☐ 7 = Divorced
☐ 8 = Other (please specify): ____________________________________

15. Other than you, how many wives does the father of the baby currently have? *[write number in box]*

☐ number of wives other than you

16. What is your religion? *[check one]*
☐ 1 = Seventh Day Adventist
☐ 2 = Nomiya
☐ 3 = Other Protestant (specify): ____________________________________
☐ 4 = Africa Independent Churches (eg: Roho, Legio Maria)
☐ 5 = Catholic
☐ 6 = Muslim
☐ 7 = Other (please specify):__________________________
☐ 8 = None
☐ 9 = Not sure

17. What is the main lighting source for your home? *[check one]*
☐ 1 = Candles / Kerosene lamps
☐ 2 = Solar power
☐ 3 = Mains electricity
☐ 4 = Other (specify):__________________________

18. What do you primarily use to cook food in your home? *[check one]*
☐ 1 = Firewood
☐ 2 = Charcoal stove (jiko)
☐ 3 = Kerosene stove
☐ 4 = Gas/electric cooker
☐ 5 = Other (specify):__________________________

19. How did you get here today? *[check all that apply]*
☐ 1 = On foot
☐ 2 = Bicycle taxi or motorcycle taxi (boda boda or piki piki)
☐ 3 = Tuk tuk
☐ 4 = Matatu
☐ 5 = Private car (taxi)
☐ 6 = Other (specify): _________________________________

20. How much did it cost to arrive here today? *[write number in box]*

			Kenya Shillings

☐ 999 = Not sure

21. About how long did it take to travel here from your home today? *[Write number in box in minutes. Eg: 2 hours = 120 minutes.]*

			Minutes

☐ 999 = Not sure

Part 2: Birth history, prenatal care and delivery
[Read] Now I am going to ask you some questions about your son, other children you may have, and your pregnancy

22. How many times have you given birth including babies who are still alive and those who have died? *[write number in box]*

		Times Given Birth

23. Of these *[number]* children, how many are/were live born sons? *[write number in box]*

		Sons

24. Of these *[number]* of sons, how many are/were circumcised? *[write number in box]*

		Circumcised

25. Did you receive any antenatal care, that is, did anyone check on your health or the health of your baby when you were pregnant with *[name]*? *[check one]*
☐ 1 = Yes *[skip a; complete b through f]*
☐ 0 = No *[complete a; skip b through f]*
☐ 2 = Not sure

a. If **NO**, why did you not receive antenatal care? *[check all that apply]*
☐ 1 = Too expensive
☐ 2 = Clinic too far away
☐ 3 = Wait is too long at the clinic
☐ 4 = No transportation
☐ 5 = Didn't need antenatal care
☐ 6 = Family/friends did not want me to go
☐ 7 = Health care worker did not want me to go
☐ 8 = Other (specify): ___________________________________

b. If **YES**, where did you receive antenatal care? *[probe to identify each location, record all mentioned]*
☐ 1 = Your home
☐ 2 = Other home

☐ 3 = Government hospital/clinic (specify): ________________________________
☐ 4 = Private hospital/clinic (specify): _____________________________________
☐ 5 = Other place (specify): _____________________________________

c. If **YES**, who provided the care? *[probe to identify each person, record all mentioned]*
☐ 1 = Doctor/Clinical Officer
☐ 2 = Nurse/Midwife
☐ 3 = Traditional Birth Attendant
☐ 4 = Community Health Worker
☐ 5 = Other (specify): _____________________________________

d. If **YES**, about how many months pregnant were you when you first received antenatal care for this pregnancy? *[Write number in box]*

		Months

☐ 99 = Not sure

e. If **YES**, about how many times did you receive antenatal care for this pregnancy? *[Write number in box]*

		Times

☐ 99 = Not sure

f. If **YES**, as part of your antenatal care during this pregnancy, were any of the following done at least once?
   i. Was your blood pressure measured? *[check one]*
   ☐ 1 = Yes
   ☐ 0 = No
   ☐ 2 = Not sure
   ii. Did you give a urine sample? *[check one]*
   ☐ 1 = Yes
   ☐ 0 = No
   ☐ 2 = Not sure
   iii. Did you give a blood sample? *[check one]*
   ☐ 1 = Yes
   ☐ 0 = No
   ☐ 2 = Not sure
   iv. Did you receive a pelvic exam? *[check one]*
   ☐ 1 = Yes
   ☐ 0 = No
   ☐ 2 = Not sure

26. Where did you give birth to your last-born son *[name]*? *[Check one. Probe to identify the location]*
☐ 1 = Your home
☐ 2 = Other home (specify): _________________________________
☐ 3 = Government hospital/clinic (specify): ________________________________
☐ 4 = Private hospital/clinic (specify): _____________________________________
☐ 5 = Other place (specify): _____________________________________

a. If **YOUR HOME** or **OTHER HOME**, what are the reasons that you did not deliver at a hospital or health care facility? *[check all that apply]*

☐ 1 = Charges at the health facility
☐ 2 = Cost of transport to the facility
☐ 3 = Unable to time the delivery - it came before I could reach the facility
☐ 4 = Comfort with the home environment
☐ 5 = Presence of family members/neighbors to support me
☐ 6 = Fear of health care workers
☐ 7 = Fear of possible mistreatment at facilities
☐ 8 = Fear of cesarean section
☐ 9 = Fear of death
☐ 10 = Told by TBA/health care provider not to go to hospital/health care facility
☐ 11 = Other (specify): ____________________________________

27. Who decided where you delivered *[name]*? *[check all that apply]*
☐ 1 = Myself
☐ 2 = The father of the baby
☐ 3 = Myself and the father equally
☐ 4 = Family member *[specify relationship to respondent]* __________________________
☐ 5 = Traditional Birth Attendant / health care provider
☐ 6 = Friends/neighbors
☐ 7 = Other (specify): ____________________________________
☐ 8 = Not sure

28. Who assisted with the delivery of your son *[name]*? Anyone else? *[Probe for the type(s) of person(s) and record all mentioned. If respondent says no one assisted, probe to determine whether any adults were present at the delivery]*
☐ 1 = Doctor/Clinical Officer
☐ 2 = Nurse/Midwife
☐ 3 = Traditional Birth Attendant
☐ 4 = Relative/Friend
☐ 5 = Other (specify): ____________________________________
☐ 6 = No one assisted

29. Was *[name]* delivered by caesarean, that is, did they cut your belly open to take the baby out? *[check one]*
☐ 1 = Yes
☐ 0 = No

30. Did you have any health problems when *[name]* was born? *[check one]*
☐ 1 = Yes
☐ 0 = No

31. Did *[name]* have any health problems when he was born? *[check one]*
☐ 1 = Yes
☐ 0 = No

Part 3: Circumcision
[Read] Now I am going to ask you some questions about male circumcision.

32. Have you ever been given information about **ADOLESCENT/ADULT** circumcision? *[check one]*
☐ 1 = Yes
☐ 0 = No

☐ 2 = Not Sure

a. If **YES**, where? *[check all that apply]*
☐ 1 = From a health care worker in the community
☐ 2 = Family member(s) (specify relationship to infant):______________________
☐ 3 = Friend(s)
☐ 4 = Radio or newspaper

☐ At a hospital/clinic: (specify):________________________________
☐ 5 = Poster or brochure
☐ 6 = Group health talk
☐ 7 = Individual consultation with a health provider
☐ 8 = Other (specify): ___________________________________

☐ 9 = Other (specify): ____
☐ 10 = Not sure

33. Have you ever been given information about **INFANT** circumcision? *[check one]*
☐ 1 = Yes
☐ 0 = No
☐ 2 = Not Sure

a. If **YES**, where? *[check all that apply]*
☐ 1 = From a health care worker in the community
☐ 2 = Family member(s) (specify relationship to infant):______________________
☐ 3 = Friend(s)
☐ 4 = Radio or newspaper

☐ At a hospital/clinic: (specify):________________________________
☐ 5 = Poster or brochure
☐ 6 = Group health talk
☐ 7 = Individual consultation with a health provider
☐ 8 = Other (specify): ___________________________________

☐ 9 = Other (specify): ____
☐ 10 = Not sure

b. If **YES**, when? *[check all that apply]*
☐ 1 = Before pregnancy
☐ 2 = During pregnancy but before delivery
☐ 3 = At delivery (or up to two days after delivery)
☐ Between delivery and now
☐ 4 = At a vaccination visit
☐ 5 = Not at a vaccination visit
☐ 6 = Today at this health facility
☐ 7 = Other (specify): ____
☐ 8 = Not sure

34. To what extent do you believe that your son *[name]* will be at risk of becoming HIV infected when he becomes a man?
☐ 1 = Not at all
☐ 2 = Not very much
☐ 3 = Somewhat

☐ 4 = Very much

35. In your opinion, what are reasons to circumcise a baby boy?
*[A: DO NOT read list of answers –**check all that apply**. Probe ➔ Any other reason?]*
*[B: Read list of answers –**check all that apply**]*

A B
☐ ☐ 1 = Protection against HIV/STI
☐ ☐ 2 = Protection against Urinary Tract Infection (UTI)
☐ ☐ 3 = Penile hygiene / cleanliness
☐ ☐ 4 = Improved cosmetic appearance of the penis
☐ ☐ 5 = Less pain than when done later
☐ ☐ 6 = It is safer than when done later
☐ ☐ 7 = Religious reason
☐ ☐ 8 = Cultural reasons
☐ ☐ 9 = There is no reason to circumcise a baby boy
☐ ☐ 10 = Not sure
☐ ☐ 11 = Other (specify): ____

36. Of those reasons, in your opinion, what is the <u>primary</u> reason to circumcise a baby boy?
*[Read respondent's answer[s] from previous question–**check only one**]*
☐ 1 = Protection against HIV/STI
☐ 2 = Protection against Urinary Tract Infection (UTI)
☐ 3 = Penile hygiene / cleanliness
☐ 4 = Improved cosmetic appearance of the penis
☐ 5 = Less pain than when done later
☐ 6 = It is safer than when done later
☐ 7 = Religious reason
☐ 8 = Cultural reasons
☐ 9 = There is no reason to circumcise a baby boy
☐ 10 = Not sure
☐ 11 = Other (specify): ____

37. In your opinion, what are reasons not to circumcise a baby boy?
*[A: DO NOT read list of answers –**check all that apply**. Probe ➔ Any other reason?]*
*[B: Read each answer –**check all that apply**]*

A B
☐ ☐ 1 = Pain
☐ ☐ 2 = Bleeding
☐ ☐ 3 = Infection
☐ ☐ 4 = Injury to the penis
☐ ☐ 5 = Death from circumcision
☐ ☐ 6 = Going against cultural tradition
☐ ☐ 7 = It is better to wait until the boy is older
☐ ☐ 8 = If the father is against it
☐ ☐ 9 = If the baby is unwell
☐ ☐ 10 = If the mother is unwell / tired after birth
☐ ☐ 11 = There is no reason not to circumcise a baby boy
☐ ☐ 12 = Not sure
☐ ☐ 13 = Other (specify): ____

38. Of those reasons, in your opinion, what is the <u>primary</u> reason not to circumcise a baby boy?
*[Read respondent's answer[s] from previous question–**check only one**]*
☐ 1 = Pain
☐ 2 = Bleeding

☐ 3 = Infection
☐ 4 = Injury to the penis
☐ 5 = Death from circumcision
☐ 6 = Going against cultural tradition
☐ 7 = It is better to wait until the boy is older
☐ 8 = If the father is against it
☐ 9 = If the baby is unwell
☐ 10 = If the mother is unwell / tired after birth
☐ 11 = There is no reason not to circumcise a baby boy
☐ 12 = Not sure
☐ 13 = Other (specify): ____

39. In your opinion, what is the best age for male circumcision? *[check all that apply]*
☐ 1 = Birth to eight weeks
☐ 2 = 9 weeks to <1 year old
☐ 3 = 1 to 9 years old
☐ 4 = 10 to 17 years old
☐ 5 = 18 years or older
☐ 6 = There is no good age for male circumcision
☐ 7 = Any age is good for male circumcision

40. In your opinion, who should decide about circumcision for a baby boy? *[check all that apply]*
☐ 1 = Mother of infant
☐ 2 = Father of infant
☐ 3 = Both parents equally
☐ 4 = Family member (specify relationship to infant): ____________________________
☐ 5 = Other (specify): _____________________________
☐ 6 = Not sure

41. If you had another baby boy, would you want him to be circumcised? *[check one]*
☐ 1 = Yes
☐ 0 = No, I would want him to remain uncircumcised.
☐ 2 = Not sure

a. If **YES**, at what age? *[check one]*
☐ 1 = Birth to eight weeks
☐ 2 = 9 weeks to <1 year old
☐ 3 = 1 to 9 years old
☐ 4 = 10 to 17 years old
☐ 5 = 18 years or older
☐ 6 = Not sure

42. In general, are you for or against a baby being circumcised before he turns two months old? *[check one]*
☐ 1 = For
☐ 0 = Against
☐ 2 = Not sure

43. Here are some faces expressing various feelings. Below each is a letter.

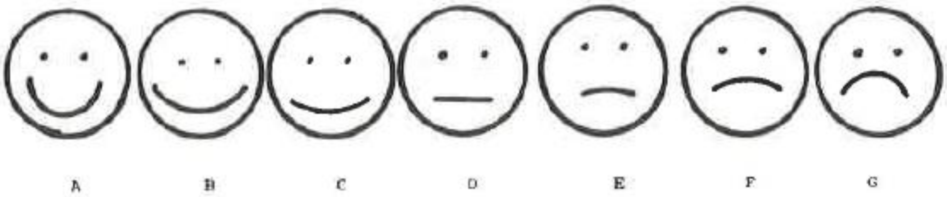

On the far left side is a mother who strongly believes that most babies should be circumcised before two months of age. On the far right side is a mother who strongly believes that most babies should ***not*** be circumcised before two months of age. In between are mothers who are less certain about circumcision for an infant before two months of age.

Which face comes closest to expressing how you feel about circumcision for a baby before two months of age?

☐ *[record letter]*

44. In general, how strongly are you **for** a baby being circumcised before two months of age?

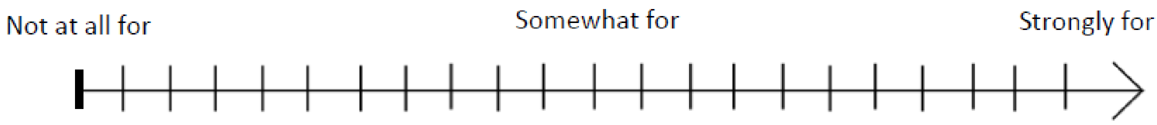

☐ *[record number]*

45. In general, how strongly are you **against** a baby being circumcised before two months of age?

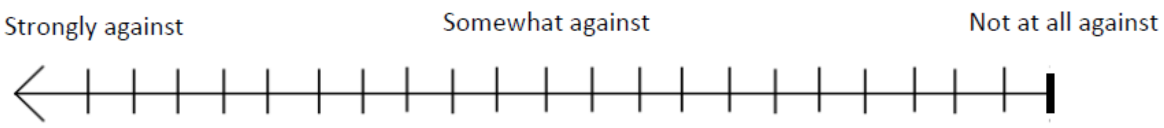

☐ *[record number]*

46. Have you ever talked to the father about circumcision for your son? *[check one]*
☐ 1 = Yes *[complete a through c and skip d]*
☐ 0 = No *[complete d only]*
☐ 2 = Not sure

a. If **YES**, when? *[check one]*
☐ 1 = Before the baby was born
☐ 2 = Around the time of delivery or just after
☐ 3 = >1 day after delivery

b. If **YES**, was he for or against circumcision generally? *[check one]*
☐ 1 = For
☐ 0 = Against

☐ 2 = Not sure

c. If **YES**, was he for or against circumcising the baby **before two months**? *[check one]*
☐ 1 = For
☐ 0 = Against
☐ 2 = Not sure

d. If **NO**, what are the reasons you did not talk about it? *[check all that apply]*
☐ 1 = I did not know circumcision was available
☐ 2 = No contact with the father
☐ 3 = I do not consult the father about the baby
☐ 4 = I did not want the boy to be circumcised
☐ 5 = Not sure
☐ 6 = Other (specify): ______________________________

47. Have you consulted anyone in deciding about whether to circumcise your son? *[check one]*
☐ 1 = Yes
☐ 0 = No

a. If **YES**, who? *[**check all that apply**]*
☐ 1 = Father of the baby
☐ 2 = Family member (specify relationship to infant): _________________________
☐ 3 = Community leader (specify): ______________________________
☐ 4 = Religious leader (specify): ______________________________
☐ 5 = Health worker (specify): ______________________________
☐ 6 = CHW
☐ 7 = Other (specify): ______________________________

48. Is your son (the baby who is here for immunization) circumcised? *[check one]*
☐ 1 = Yes he is circumcised [*Go to Question 49*]
☐ 0 = No, he is not circumcised [***Skip to Question 58 (if baby <=2 months) or 60 (if baby > 2 months)***]

Circumcision = YES

49. When was the baby circumcised? *[dd/mm/yy]* ______/______/______

50. Where was he circumcised?
☐ 1 = In the home
☐ 2 = At a health clinic (specify): ______________________________
☐ 3 = Other place (specify): ______________________________

51. Who performed the circumcision?
☐ 1 = Clinician
☐ 2 = Traditional circumciser
☐ 3 = Religious leader
☐ 4 = Other (specify): ______________________________

52. Were you for or against circumcising the baby?
☐ 1 = For
☐ 0 = Against

☐ 2 = Not sure

53. Who was the primary person who made the decision to circumcise your son? *[**check only one**]*
☐ 1 = Myself
☐ 2 = Father of infant
☐ 3 = Both parents equally
☐ 4 = Family member (specify relationship to infant): ______________________________
☐ 5 = Other (specify): ________________________________
☐ 6 = Not sure

a. According to the primary decision maker, what were the reasons for choosing circumcision?
*[**A:** DO NOT read list of answers –**check all that apply**. Probe ➔ Any other reason?]*
*[**B:** Read list of answers –**check all that apply**]*
A B
☐ ☐ 1 = Protection against HIV/STI
☐ ☐ 2 = Protection against Urinary Tract Infection (UTI)
☐ ☐ 3 = Penile hygiene / cleanliness
☐ ☐ 4 = Improved cosmetic appearance of the penis
☐ ☐ 5 = Less pain than when done later
☐ ☐ 6 = It is safer than when done later
☐ ☐ 7 = Religious reason
☐ ☐ 8 = Cultural reason
☐ ☐ 9 = Not sure
☐ ☐ 10 = Other (specify): ____

b. According to the primary decision maker, what was the single most important reason for choosing to circumcise the baby? *[Read respondent's answer[s] from previous question–**check only one**]*
☐ 1 = Protection against HIV/STI
☐ 2 = Protection against Urinary Tract Infection (UTI)
☐ 3 = Penile hygiene / cleanliness
☐ 4 = Improved cosmetic appearance of the penis
☐ 5 = Less pain than when done later
☐ 6 = It is safer than when done later
☐ 7 = Religious reason
☐ 8 = Cultural reason
☐ 9 = Not sure
☐ 10 = Other (specify): ____

54. So far, how satisfied or dissatisfied are you with the circumcision of your baby? *[check one]*
☐ 1 = Satisfied
☐ 2 = Neither satisfied nor dissatisfied
☐ 3 = Dissatisfied

a. If **DISSATISFIED**, what would make you more satisfied?

____

55. If you could do it again, would you circumcise your baby? *[check one]*
☐ 1 = Yes
☐ 0 = No
☐ 2 = Not sure

a. Would you prefer that your baby is circumcised at home or in a health facility?
☐ 1 = At home
☐ 0 = At a health facility
☐ 2 = Not sure

56. Would you recommend infant circumcision to a friend or relative? *[check one]*
☐ 1 = Yes
☐ 0 = No
☐ 2 = Not sure

57. May I inspect the circumcision of your son to be sure that he was circumcised properly? *[check one]*
☐ 1 = Yes
☐ 0 = No

a. *[For interviewer: If **YES**, What is the circumcision status of the child?]*
☐ 1 = Circumcised
☐ 0 = Uncircumcised
☐ 2 = Partially circumcised
☐ 3 = Not sure

b. *[For interviewer: If **YES**, How would you rate the healing from the circumcision?]*
☐ 1 = Healing appears normal
☐ 2 = Healing does not appear normal (specify): ______________________________
☐ 3 = Not sure

Circumcision = NO
AND
Baby age ≤2 months

58. If we offered circumcision for your baby today, would you take it up? *[check one]*
☐ 1 = Yes
☐ 0 = No
☐ 3 = Not sure

a. Why?
*[**A**: DO NOT read list of answers –**check all that apply**. Probe ➔ Any other reason?]*
*[**B**: Read list of answers –**check all that apply**]*
A **B**
☐ ☐ 1 = Need to consult the father
☐ ☐ 2 = Need more information about circumcision

☐ ☐ 3 = Pain
☐ ☐ 4 = Bleeding
☐ ☐ 5 = Infection
☐ ☐ 6 = Injury to the penis
☐ ☐ 7 = Death from circumcision
☐ ☐ 8 = Going against cultural tradition
☐ ☐ 9 = It is better to wait until the boy is older
☐ ☐ 10 = The father is against it

☐ ☐ 11 = The baby is unwell
☐ ☐ 12 = I am unwell / tired
☐ ☐ 13 = There is no reason to circumcise a baby boy

☐ ☐ 14 = Protection against HIV/STI
☐ ☐ 15 = Protection against Urinary Tract Infection (UTI)
☐ ☐ 16 = Penile hygiene / cleanliness
☐ ☐ 17 = Improved cosmetic appearance of the penis
☐ ☐ 18 = Less pain / the procedure is safer when done earlier
☐ ☐ 19 = Religious reason

☐ ☐ 20 = Not sure
☐ ☐ 21 = Other (specify): ____

b. Of those reasons, which is the primary reason you would/would not take up circumcision? *[Read respondent's answer[s] from previous question–**check only one**]*
☐ 1 = Need to consult the father
☐ 2 = Need more information about circumcision

☐ 3 = Pain
☐ 4 = Bleeding
☐ 5 = Infection
☐ 6 = Injury to the penis
☐ 7 = Death from circumcision
☐ 8 = Going against cultural tradition
☐ 9 = It is better to wait until the boy is older
☐ 10 = The father is against it
☐ 11 = The baby is unwell
☐ 12 = I am unwell / tired
☐ 13 = There is no reason to circumcise a baby boy

☐ 14 = Protection against HIV/STI
☐ 15 = Protection against Urinary Tract Infection (UTI)
☐ 16 = Penile hygiene / cleanliness
☐ 17 = Improved cosmetic appearance of the penis
☐ 18 = Less pain / the procedure is safer when done earlier
☐ 19 = Religious reason

☐ 20 = Not sure
☐ 21 = Other (specify): ____

59. If it were only up to you, how likely is it you would circumcise your baby before two months of age? *[check one]*
☐ 1 = Unlikely
☐ 2 = Neither likely nor unlikely
☐ 3 = Likely
☐ 4 = Not sure

a. If your baby were to be circumcised, would you prefer that your baby is circumcised at home or in a health facility? *[check one]*
☐ 1 = At home
☐ 0 = At a health facility
☐ 2 = Not sure

Circumcision = NO
AND
Baby age >2 months

60. Has anyone told you infant circumcision services are available?
☐ 1 = Yes
☐ 0 = No

a. *[If **NO**]* Circumcision is only available to babies before two months of age. If we had offered circumcision for your baby before he turned two months, how likely is it you would have taken it up? *[check one]*
☐ 1 = Unlikely
☐ 2 = Neither likely nor unlikely
☐ 3 = Likely
☐ 4 = Not sure

i. Why?
*[**A**: DO NOT read list of answers –**check all that apply**. Probe ➔ Any other reason?]*
*[**B**: Read list of answers –**check all that apply**]*
A B
☐ ☐ 1 = Need to consult the father
☐ ☐ 2 = Need more information about circumcision

☐ ☐ 3 = Pain
☐ ☐ 4 = Bleeding
☐ ☐ 5 = Infection
☐ ☐ 6 = Injury to the penis
☐ ☐ 7 = Death from circumcision
☐ ☐ 8 = Going against cultural tradition
☐ ☐ 9 = It is better to wait until the boy is older
☐ ☐ 10 = The father is against it
☐ ☐ 11 = The baby is unwell
☐ ☐ 12 = I am unwell / tired
☐ ☐ 13 = There is no reason to circumcise a baby boy

☐ ☐ 14 = Protection against HIV/STI
☐ ☐ 15 = Protection against Urinary Tract Infection (UTI)
☐ ☐ 16 = Penile hygiene / cleanliness
☐ ☐ 17 = Improved cosmetic appearance of the penis
☐ ☐ 18 = Less pain / the procedure is safer when done earlier
☐ ☐ 19 = Religious reason

☐ ☐ 20 = Not sure
☐ ☐ 21 = Other (specify): ____

ii. Of those reasons, which is the primary reason for how likely/unlikely you would have been to take up circumcision? *[Read respondent's answer[s] from the previous question –**check only one**]*
☐ 1 = Need to consult the father
☐ 2 = Need more information about circumcision

☐ 3 = Pain

☐ 4 = Bleeding
☐ 5 = Infection
☐ 6 = Injury to the penis
☐ 7 = Death from circumcision
☐ 8 = Going against cultural tradition
☐ 9 = It is better to wait until the boy is older
☐ 10 = The father is against it
☐ 11 = The baby is unwell
☐ 12 = I am unwell / tired
☐ 13 = There is no reason to circumcise a baby boy

☐ 14 = Protection against HIV/STI
☐ 15 = Protection against Urinary Tract Infection (UTI)
☐ 16 = Penile hygiene / cleanliness
☐ 17 = Improved cosmetic appearance of the penis
☐ 18 = Less pain / the procedure is safer when done earlier
☐ 19 = Religious reason

☐ 20 = Not sure
☐ 21 = Other (specify): ____

b. *[If **YES**]* Were you for or against him being circumcised before two months of age?
☐ 1 = For
☐ 0 = Against
☐ 2 = Not sure

i. Why?
*[**A**: DO NOT read list of answers –**check all that apply**. Probe ➔ Any other reason?]*
*[**B**: Read list of answers –**check all that apply**]*
A **B**
☐ ☐ 1 = Need to consult the father
☐ ☐ 2 = Need more information about circumcision

☐ ☐ 3 = Pain
☐ ☐ 4 = Bleeding
☐ ☐ 5 = Infection
☐ ☐ 6 = Injury to the penis
☐ ☐ 7 = Death from circumcision
☐ ☐ 8 = Going against cultural tradition
☐ ☐ 9 = It is better to wait until the boy is older
☐ ☐ 10 = The father is against it
☐ ☐ 11 = The baby is unwell
☐ ☐ 12 = I am unwell / tired
☐ ☐ 13 = There is no reason to circumcise a baby boy

☐ ☐ 14 = Protection against HIV/STI
☐ ☐ 15 = Protection against Urinary Tract Infection (UTI)
☐ ☐ 16 = Penile hygiene / cleanliness
☐ ☐ 17 = Improved cosmetic appearance of the penis
☐ ☐ 18 = Less pain / the procedure is safer when done earlier
☐ ☐ 19 = Religious reason

☐ ☐ 20 = Not sure

☐ ☐ 21 = Other (specify): ______________________________________

ii. Of those reasons, which is the <u>primary</u> reason for your opinion about circumcision for your son? *[DO NOT read list of answers –**check only one**]*
☐ 1 = Need to consult the father
☐ 2 = Need more information about circumcision

☐ 3 = Pain
☐ 4 = Bleeding
☐ 5 = Infection
☐ 6 = Injury to the penis
☐ 7 = Death from circumcision
☐ 8 = Going against cultural tradition
☐ 9 = It is better to wait until the boy is older
☐ 10 = The father is against it
☐ 11 = The baby is unwell
☐ 12 = I am unwell / tired
☐ 13 = There is no reason to circumcise a baby boy

☐ 14 = Protection against HIV/STI
☐ 15 = Protection against Urinary Tract Infection (UTI)
☐ 16 = Penile hygiene / cleanliness
☐ 17 = Improved cosmetic appearance of the penis
☐ 18 = Less pain / the procedure is safer when done earlier
☐ 19 = Religious reason

☐ 20 = Not sure
☐ 21 = Other (specify): ____

61. If your baby were less than 60 days old and you had an opportunity to circumcise, would you have him circumcised? *[check one]*
☐ 1 = Yes
☐ 0 = No
☐ 2 = Not sure

Part 4: Beliefs about Circumcision
[Read] Now I am going to ask you some questions about your beliefs about male circumcision.

62. It easier to keep a penis clean if a man is . . . . ? *[check one]*
☐ 1 = Circumcised
☐ 2 = Uncircumcised
☐ 3 = No difference
☐ 4 = Not sure

63. It easier for a man to get a disease from a woman if the man is. . . . ? *[check one]*
☐ 1 = Circumcised
☐ 2 = Uncircumcised
☐ 3 = No difference
☐ 4 = Not sure

64. It is easier for a man to get AIDS if he is. . . . ? *[check one]*
☐ 1 = Circumcised
☐ 2 = Uncircumcised

☐ 3 = No difference
☐ 4 = Not sure

65. Men enjoy sex more if they are. . . . ? *[check one]*
☐ 1 = Circumcised
☐ 2 = Uncircumcised
☐ 3 = No difference
☐ 4 = Not sure

66. Most women enjoy sex more with a man who is. . . . ? *[check one]*
☐ 1 = Circumcised
☐ 2 = Uncircumcised
☐ 3 = No difference
☐ 4 = Not sure

67. Men are more promiscuous if they are. . . . *[check one]*
☐ 1 = Circumcised
☐ 2 = Uncircumcised
☐ 3 = No difference
☐ 4 = Not sure

68. A penis looks better if it is . . . . ? *[check one]*
☐ 1 = Circumcised
☐ 2 = Uncircumcised
☐ 3 = No difference
☐ 4 = Not sure

69. In the past month, how many shillings have you earned from all sources? *[check one]*
☐ 1 = None
☐ 2 = < 2000
☐ 3 = 2000-4999
☐ 4 = 5000-9999
☐ 5 = 10000-25000
☐ 6 = > 25000

70. Have you ever talked with the father of the baby about his HIV status? *[check one]*
☐ 1 = Yes
☐ 0 = No
☐ 2 = Not sure
☐ 3 = Refused to answer

71. Have you ever been tested for HIV? *[check one]*
☐ 1 = Yes
☐ 0 = No
☐ 2 = Not sure
☐ 3 = Refused to answer

   a. If **YES**, were you tested during pregnancy/since you gave birth? *[check one]*
   ☐ 1 = Yes
   ☐ 0 = No
   ☐ 2 = Not sure
   ☐ 3 = Refused to answer

   b. If **YES**, are you willing to give your most recent results? *[check one]*

☐ 1 = Yes
☐ 0 = No

i. If **YES**, what were they? *[check one]*
☐ 1 = Positive
☐ 0 = Negative
☐ 2 = Not sure

72. Whether you have been tested or not, in your opinion, what is your HIV status? *[check one]*
☐ 1 = HIV positive
☐ 0 = HIV negative
☐ 2 = Not sure
☐ 3 = Refused to answer

73. What do you think the HIV status of the father of the baby is? *[check one]*
☐ 1 = HIV positive
☐ 0 = HIV negative
☐ 2 = Not sure
☐ 3 = Refused to answer

74. Please provide a contact number or contact information, in case we need to ask you any follow-up questions
    b. Phone number: ____

    c. Phone owner (name): ____

    d. Other contact information: ____

75. **If you provided consent for us to contact the father of the baby**, please give a contact number or contact information for the father, so we can reach him for an interview *[Check the consent form to confirm whether the participant gave consent to contact the father of the infant.]*

    e. Phone number: ____

    f. Phone owner (name): ____

    g. Other contact information: ____

[Read: Thank you for your time. Those are all the questions I have for you. Do you have any questions for me right now?]

[To be answered by the interviewer:]

76. *Primary language of interview*
☐ 1 = English
☐ 2 = DhoLuo
☐ 3 = Kiswahili

77. *End Time*

:

78. *Interviewer code*

79. *Notes:*
