# Supplementary material for: Prospective comparison of two models of integrating early infant male circumcision with maternal child health services in Kenya: The Mtoto Msafi Mbili Study
Source: PLoS One. 2017 Sep 7;12(9):e0184170. doi: 10.1371/journal.pone.0184170 (PMC5589171; doi:10.1371/journal.pone.0184170)
Supplement: S3 File — (PDF) [file pone.0184170.s003.pdf]

MTOTO MSAFI MBILI	Questionnaire - Father
Date (dd/mm/yy): ____/____/____	Participant Number: ________________________<br><br>Site ID:_________

[To be answered by the interviewer:]

1. *Start Time*

:

2. *[If interview taking place at home] Record geographic coordinates of location*

o	’	”	
			S

o	’	”	
			E

Part 1: Demographic Information
[Read] I am going to start by asking you some questions about yourself, the baby and the mother of the baby. Are you ready to begin?

3. What is your date of birth? *[dd/mm/yy]* ______/______/______

4. How old are you now? ___________ years old

5. What is your son’s date of birth? *[dd/mm/yy]* ______/______/______

   [To be answered by the interviewer:]
   a. *Son receiving OPV I is:*
      ☐ 1 = ≤ 2 months old
      ☐ 0 = > 2 months old

6. What is your Ethnic origin? *[check one]*
   ☐ 1 = Luo
   ☐ 0 = Other (specify): _________________________________

7. What is the Ethnic origin of the mother of the baby? *[check one]*
   ☐ 1 = Luo
   ☐ 0 = Other (specify): _________________________________
   ☐ 2 = Not sure

8. What is your circumcision status? *[check one]*
   ☐ 1 = Circumcised
   ☐ 0 = Uncircumcised

☐ 2 = Not sure

a. If **CIRCUMCISED**, when were you circumcised? *[check one]*
   ☐ 1 = Birth to eight weeks
   ☐ 2 = >8 weeks to <1 year old
   ☐ 3 = 1 to 9 years old
   ☐ 4 = 10 to 17 years old
   ☐ 5 = 18 to 29 years old
   ☐ 6 = 30 years or older

b. If **CIRCUMCISED**, who circumcised you? *[check one]*
   ☐ 1 = A clinician
   ☐ 2 = A traditional circumciser
   ☐ 3 = Not sure
   ☐ 4 = Other (specify): ______________________________

9. What district do you currently reside in (spend the most nights sleeping in)? *[check one]*
   ☐ 1 = Rachuonyo North
   ☐ 2 = Rachuonyo South
   ☐ 3 = Other (specify): ______________________________

10. What is the highest level of school you completed? *[check one]*
   ☐ 0 = No level completed

   ☐ 1 = Class 1
   ☐ 2 = Class 2
   ☐ 3 = Class 3
   ☐ 4 = Class 4
   ☐ 5 = Class 5
   ☐ 6 = Class 6
   ☐ 7 = Class 7
   ☐ 8 = Class 8

   ☐ 9 = Form 1
   ☐ 10 = Form 2
   ☐ 11 = Form 3
   ☐ 12 = Form 4

   ☐ 13 = Post-graduate Certificate
   ☐ 14 = Post-graduate Diploma
   ☐ 15 = Post-graduate Degree

11. Are you currently employed? *[check one]*
   ☐ 1 = Yes
   ☐ 0 = No

12. What is your primary occupation? *[check one]*
   ☐ 1 = Hawker/Small Business owner
   ☐ 2 = Farmer
   ☐ 3 = Professional/Managerial
   ☐ 4 = Student
   ☐ 5 = Other (specify):____________________________________

13. What is your current marital status? *[check one]*

☐ 1 = Not legally married, without a regular live-in partner
☐ 2 = Not legally married, with a regular live-in partner
☐ 3 = Legally married, not living with wife
☐ 4 = Legally married, living with wife
☐ 5 = Separated
☐ 6 = Widower
☐ 7 = Divorced
☐ 8 = Other (please specify): ___________________________________

14. What is your religion? *[check one]*
☐ 1 = Seventh Day Adventist
☐ 2 = Nomiya
☐ 3 = Other Protestant (specify): ___________________________________
☐ 4 = Africa Independent Churches (eg: Roho, Legio Maria)
☐ 5 = Catholic
☐ 6 = Muslim
☐ 7 = Other (please specify):_________________________
☐ 8 = None
☐ 9 = Not sure

15. What is the main lighting source for your home? *[check one]*
☐ 1 = Candles / Kerosene lamps
☐ 2 = Solar power
☐ 3 = Mains electricity
☐ 4 = Other (specify):_________________________

16. What do you primarily use to cook food in your home? *[check one]*
☐ 1 = Firewood
☐ 2 = Charcoal stove (jiko)
☐ 3 = Kerosene stove
☐ 4 = Gas/electric cooker
☐ 5 = Other (specify):_________________________

Part 2: Circumcision
[Read] Now I am going to ask you some questions about male circumcision.

17. Have you ever been given information about **ADOLESCENT/ADULT** circumcision? *[check one]*
☐ 1 = Yes
☐ 0 = No
☐ 2 = Not Sure

a. If **YES**, where? *[check all that apply]*
☐ 1 = From a health care worker in the community
☐ 2 = Family member(s) (specify relationship to infant):_____________________
☐ 3 = Friend(s)
☐ 4 = Radio or newspaper

☐ At a hospital/clinic: (specify):______________________________
☐ 5 = Poster or brochure
☐ 6 = Group health talk
☐ 7 = Individual consultation with a health provider

☐ 8 = Other (specify): ____

☐ 9 = Community event (specify): ______________________________________

☐ 10 = Other (specify): ____

☐ 11 = Not sure

18. Have you ever been given information about **INFANT** circumcision? *[check one]*

☐ 1 = Yes

☐ 0 = No

☐ 2 = Not Sure

a. If **YES**, where? *[check all that apply]*

☐ 1 = From a health care worker in the community

☐ 2 = Family member(s) (specify relationship to infant):_____________________

☐ 3 = Friend(s)

☐ 4 = Radio or newspaper

☐ At a hospital/clinic: (specify):______________________________

☐ 5 = Poster or brochure

☐ 6 = Group health talk

☐ 7 = Individual consultation with a health provider

☐ 8 = Other (specify): ____

☐ 9 = Community event (specify): ______________________________________

☐ 10 = Other (specify): ____

☐ 11 = Not sure

b. If **YES**, when? *[check all that apply]*

☐ 1 = Before your wife was most recently pregnant

☐ 2 = During your wife's most recent pregnancy but before delivery

☐ 3 = At delivery (or up to two days after delivery)

☐ Between delivery and now

☐ 4 = At a vaccination visit

☐ 5 = Not at a vaccination visit

☐ 6 = Today at this health facility

☐ 7 = Other (specify): ____

☐ 8 = Not sure

19. In your opinion, what are reasons to circumcise a baby boy?

*[**A**: DO NOT read list of answers –**check all that apply**. Probe ➔ Any other reason?]*

*[**B**: Read list of answers –**check all that apply**]*

A	*B*
☐	☐ 1 = Protection against HIV/STI
☐	☐ 2 = Protection against Urinary Tract Infection (UTI)
☐	☐ 3 = Penile hygiene / cleanliness
☐	☐ 4 = Improved cosmetic appearance of the penis
☐	☐ 5 = Less pain than when done later
☐	☐ 6 = It is safer than when done later
☐	☐ 7 = Religious reason
☐	☐ 8 = Cultural reasons
☐	☐ 9 = There is no reason to circumcise a baby boy
☐	☐ 10 = Not sure
☐	☐ 11 = Other (specify): ____

20. Of those reasons, in your opinion, what is the <u>primary</u> reason to circumcise a baby boy?
*[Read respondent's answer[s] from previous question–**check only one**]*
☐ 1 = Protection against HIV/STI
☐ 2 = Protection against Urinary Tract Infection (UTI)
☐ 3 = Penile hygiene / cleanliness
☐ 4 = Improved cosmetic appearance of the penis
☐ 5 = Less pain than when done later
☐ 6 = It is safer than when done later
☐ 7 = Religious reason
☐ 8 = Cultural reasons
☐ 9 = There is no reason to circumcise a baby boy
☐ 10 = Not sure
☐ 11 = Other (specify): ____

21. In your opinion, what are reasons not to circumcise a baby boy?
*[**A:** DO NOT read list of answers –**check all that apply**. Probe ➔ Any other reason?]*
*[**B**: Read list of answers –**check all that apply**]*
A ***B***
☐ ☐ 1 = Pain
☐ ☐ 2 = Bleeding
☐ ☐ 3 = Infection
☐ ☐ 4 = Injury to the penis
☐ ☐ 5 = Death from circumcision
☐ ☐ 6 = Going against cultural tradition
☐ ☐ 7 = It is better to wait until the boy is older
☐ ☐ 8 = If the mother is against it
☐ ☐ 9 = If the baby is unwell
☐ ☐ 10 = If the mother is unwell / tired after birth
☐ ☐ 11 = There is no reason not to circumcise a baby boy
☐ ☐ 12 = Not sure
☐ ☐ 13 = Other (specify): ____

22. Of those reasons, in your opinion, what is the <u>primary</u> reason not to circumcise a baby boy?
*[Read respondent's answer[s] from previous question–**check only one**]*
☐ 1 = Pain
☐ 2 = Bleeding
☐ 3 = Infection
☐ 4 = Injury to the penis
☐ 5 = Death from circumcision
☐ 6 = Going against cultural tradition
☐ 7 = It is better to wait until the boy is older
☐ 8 = If the mother is against it
☐ 9 = If the baby is unwell
☐ 10 = If the mother is unwell / tired after birth
☐ 11 = There is no reason not to circumcise a baby boy
☐ 12 = Not sure
☐ 13 = Other (specify): ____

23. In your opinion, what is the best age for male circumcision? *[check all that apply]*
☐ 1 = Birth to eight weeks
☐ 2 = 9 weeks to <1 year old
☐ 3 = 1 to 9 years old
☐ 4 = 10 to 17 years old
☐ 5 = 18 years or older

☐ 6 = There is no good age for male circumcision
☐ 7 = Any age is good for male circumcision

24. If you had another baby boy, would you want him to be circumcised? *[check one]*
☐ 1 = Yes
☐ 0 = No, I would want him to remain uncircumcised.
☐ 2 = Not sure

a. If **YES**, at what age? *[check one]*
☐ 1 = Birth to eight weeks
☐ 2 = 9 weeks to <1 year old
☐ 3 = 1 to 9 years old
☐ 4 = 10 to 17 years old
☐ 5 = 18 years or older
☐ 6 = Not sure

25. In general, are you for or against a baby being circumcised before he turns two months old?
☐ 1 = For
☐ 0 = Against
☐ 2 = Not sure

26. Here are some faces expressing various feelings. Below each is a letter.

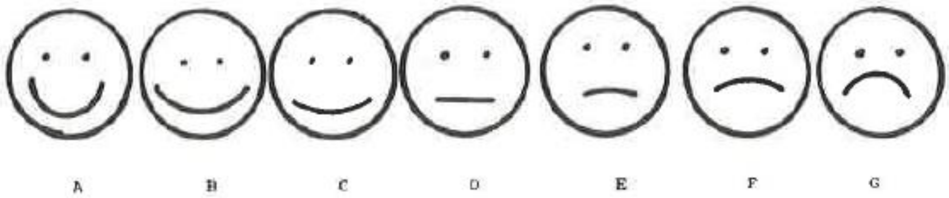

Which face comes closest to expressing how you feel about circumcision for a baby before two months of age?

☐ *[record letter]*

27. In general, how strongly are you **for** a baby being circumcised before two months of age?

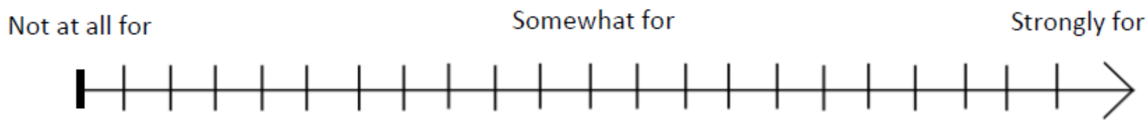

☐ *[record number]*

28. In general, how strongly are you **against** a baby being circumcised before two months of age?

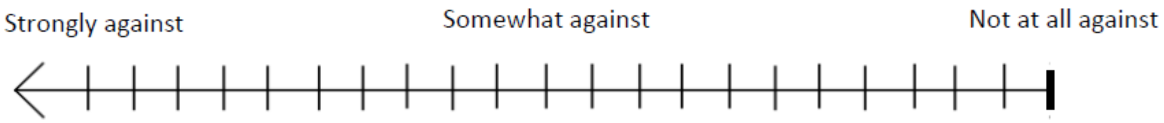

☐ *[record number]*

29. Have you ever talked to the mother about circumcision for your son? *[check one]*
    ☐ 1 = Yes *[complete a through c and skip d]*
    ☐ 0 = No *[complete d only]*
    ☐ 2 = Not sure

    a. If **YES**, when? *[check one]*
       ☐ 1 = Before the baby was born
       ☐ 2 = Around the time of delivery or just after
       ☐ 3 = >1 day after delivery

    b. If **YES**, was she for or against circumcision? *[check one]*
       ☐ 1 = For
       ☐ 0 = Against
       ☐ 2 = Not sure

    c. If **YES**, was she for or against circumcising the baby **before two months of age**? *[check one]*
       ☐ 1 = For
       ☐ 0 = Against
       ☐ 2 = Not sure

    d. If **NO**, what are the reasons you did not talk about it? *[check all that apply]*
       ☐ 1 = I did not know circumcision was available
       ☐ 2 = No contact with the mother
       ☐ 3 = I do not consult the mother about the baby
       ☐ 4 = I did not want the boy to be circumcised
       ☐ 5 = Not sure
       ☐ 6 = Other (specify): ______________________________

30. Have you consulted anyone in deciding about whether to circumcise your son? *[check one]*
    ☐ 1 = Yes
    ☐ 0 = No

    a. If **YES**, who? *[**check all that apply**]*
       ☐ 1 = Mother of the baby
       ☐ 2 = Family member (specify relationship to infant): _________________________
       ☐ 3 = Community leader (specify): ______________________________
       ☐ 4 = Religious leader (specify): ______________________________
       ☐ 5 = Health worker (specify): ______________________________
       ☐ 6 = CHW
       ☐ 7 = Other (specify): ______________________________

31. Is your son (the baby who went for immunization) circumcised? *[check one]*
    ☐ 1 = Yes he is circumcised [*Go to Question 31*]

☐ 0 = No, he is not circumcised [***Skip to Question 39 (if baby ≤2 months old) or Question 41 (if baby >2 months)***]

Circumcision = YES

32. When was the baby circumcised? *[dd/mm/yy]* ______/______/______

33. Where was he circumcised?
☐ 1 = In the home
☐ 2 = At a health clinic (specify): ______________________________
☐ 3 = Other place (specify): ______________________________

34. Who performed the circumcision?
☐ 1 = Clinician
☐ 2 = Traditional circumciser
☐ 3 = Religious leader
☐ 4 = Other (specify): ______________________________

35. Were you for or against circumcising the baby?
☐ 1 = For
☐ 0 = Against
☐ 2 = Not sure

36. Who was the primary person who made the decision to circumcise your son? *[**check only one**]*
☐ 1 = Myself
☐ 2 = Mother of infant
☐ 3 = Both parents equally
☐ 4 = Family member (specify relationship to infant): ____________________________
☐ 5 = Other (specify): ______________________________
☐ 6 = Not sure

a. According to the primary decision maker, what were the reasons for choosing circumcision?
*[**A**: DO NOT read list of answers –**check all that apply**. Probe ➔ Any other reason?]*
*[**B**: Read list of answers –**check all that apply**]*

A	***B***
☐	☐ 1 = Protection against HIV/STI
☐	☐ 2 = Protection against Urinary Tract Infection (UTI)
☐	☐ 3 = Penile hygiene / cleanliness
☐	☐ 4 = Improved cosmetic appearance of the penis
☐	☐ 5 = Less pain than when done later
☐	☐ 6 = It is safer than when done later
☐	☐ 7 = Religious reason
☐	☐ 8 = Cultural reason
☐	☐ 9 = Not sure
☐	☐ 10 = Other (specify): ____

b. According to the primary decision maker, what was the single most important reason for choosing to circumcise the baby? *[Read respondent's answer[s] from previous question–**check only one**]*

☐ 1 = Protection against HIV/STI
☐ 2 = Protection against Urinary Tract Infection (UTI)
☐ 3 = Penile hygiene / cleanliness
☐ 4 = Improved cosmetic appearance of the penis
☐ 5 = Less pain than when done later
☐ 6 = It is safer than when done later
☐ 7 = Religious reason
☐ 8 = Cultural reason
☐ 9 = Not sure
☐ 10 = Other (specify): ____

37. So far, how satisfied or dissatisfied are you with the circumcision of your baby? *[check one]*
☐ 1 = Satisfied
☐ 2 = Neither satisfied nor dissatisfied
☐ 3 = Dissatisfied

a. If **DISSATISFIED**, what would make you more satisfied?

____

38. If you could do it again, would you circumcise your baby? *[check one]*
☐ 1 = Yes
☐ 0 = No
☐ 2 = Not sure

39. Would you recommend infant circumcision to a friend or relative? *[check one]*
☐ 1 = Yes
☐ 0 = No
☐ 2 = Not sure

Circumcision = NO
AND
Baby age ≤2 months

40. If we offered circumcision for your baby today, would you take it up? *[check one]*
☐ 1 = Yes
☐ 0 = No
☐ 2 = Not sure

a. Why?
*[**A:** DO NOT read list of answers –**check all that apply**. Probe ➔ Any other reason?]*
*[**B**: Read list of answers –**check all that apply**]*
A ***B***
☐ ☐ 1 = Need to consult the mother
☐ ☐ 2 = Need more information about circumcision

☐ ☐ 3 = Pain
☐ ☐ 4 = Bleeding
☐ ☐ 5 = Infection
☐ ☐ 6 = Injury to the penis
☐ ☐ 7 = Death from circumcision

☐ ☐ 8 = Going against cultural tradition
☐ ☐ 9 = It is better to wait until the boy is older
☐ ☐ 10 = The mother is against it
☐ ☐ 11 = The baby is unwell
☐ ☐ 12 = I am unwell / tired
☐ ☐ 13 = There is no reason to circumcise a baby boy

☐ ☐ 14 = Protection against HIV/STI
☐ ☐ 15 = Protection against Urinary Tract Infection (UTI)
☐ ☐ 16 = Penile hygiene / cleanliness
☐ ☐ 17 = Improved cosmetic appearance of the penis
☐ ☐ 18 = Less pain / the procedure is safer when done earlier
☐ ☐ 19 = Religious reason

☐ ☐ 20 = Not sure
☐ ☐ 21 = Other (specify): ____

b. Of those reasons, which is the primary reason for why you would/would not take up circumcision? *[Read respondent's answer[s] from previous question–* ***check only one****]*
☐ 1 = Need to consult the mother
☐ 2 = Need more information about circumcision

☐ 3 = Pain
☐ 4 = Bleeding
☐ 5 = Infection
☐ 6 = Injury to the penis
☐ 7 = Death from circumcision
☐ 8 = Going against cultural tradition
☐ 9 = It is better to wait until the boy is older
☐ 10 = The mother is against it
☐ 11 = The baby is unwell
☐ 12 = I am unwell / tired
☐ 13 = There is no reason to circumcise a baby boy

☐ 14 = Protection against HIV/STI
☐ 15 = Protection against Urinary Tract Infection (UTI)
☐ 16 = Penile hygiene / cleanliness
☐ 17 = Improved cosmetic appearance of the penis
☐ 18 = Less pain / the procedure is safer when done earlier
☐ 19 = Religious reason

☐ 20 = Not sure
☐ 21 = Other (specify): ____

41. If it were only up to you, how likely is it you would circumcise your baby before two months of age? *[check one]*
☐ 1 = Unlikely
☐ 2 = Neither likely nor unlikely
☐ 3 = Likely
☐ 4 = Not sure

Circumcision = NO

AND
Baby age >2 months

42. Has anyone told you infant circumcision services are available?
    ☐ 1 = Yes
    ☐ 0 = No

    a. *[If **NO**]* Circumcision is only available to babies before two months of age. If we had offered circumcision for your baby before he turned two months, how likely is it you would have taken it up? *[check one]*
       ☐ 1 = Unlikely
       ☐ 2 = Neither likely nor unlikely
       ☐ 3 = Likely
       ☐ 4 = Not sure

       i. Why?
       *[**A:** DO NOT read list of answers –**check all that apply**. Probe ➔ Any other reason?]*
       *[**B**: Read list of answers –**check all that apply**]*

A	***B***
☐	☐ 1 = Need to consult the mother
☐	☐ 2 = Need more information about circumcision

☐	☐ 3 = Pain
☐	☐ 4 = Bleeding
☐	☐ 5 = Infection
☐	☐ 6 = Injury to the penis
☐	☐ 7 = Death from circumcision
☐	☐ 8 = Going against cultural tradition
☐	☐ 9 = It is better to wait until the boy is older
☐	☐ 10 = The mother is against it
☐	☐ 11 = The baby is unwell
☐	☐ 12 = The mother is unwell / tired
☐	☐ 13 = There is no reason to circumcise a baby boy

☐	☐ 14 = Protection against HIV/STI
☐	☐ 15 = Protection against Urinary Tract Infection (UTI)
☐	☐ 16 = Penile hygiene / cleanliness
☐	☐ 17 = Improved cosmetic appearance of the penis
☐	☐ 18 = Less pain / the procedure is safer when done earlier
☐	☐ 19 = Religious reason

☐	☐ 20 = Not sure
☐	☐ 21 = Other (specify):

____

       ii. Of those reasons, which is the <u>primary</u> reason for how likely/unlikely you would have been to take up circumcision? *[Read respondent's answer[s] from previous question –**check only one**]*
       ☐ 1 = Need to consult the mother
       ☐ 2 = Need more information about circumcision

       ☐ 3 = Pain

☐ 4 = Bleeding
☐ 5 = Infection
☐ 6 = Injury to the penis
☐ 7 = Death from circumcision
☐ 8 = Going against cultural tradition
☐ 9 = It is better to wait until the boy is older
☐ 10 = The mother is against it
☐ 11 = The baby is unwell
☐ 12 = The mother is unwell / tired
☐ 13 = There is no reason to circumcise a baby boy

☐ 14 = Protection against HIV/STI
☐ 15 = Protection against Urinary Tract Infection (UTI)
☐ 16 = Penile hygiene / cleanliness
☐ 17 = Improved cosmetic appearance of the penis
☐ 18 = Less pain / the procedure is safer when done earlier
☐ 19 = Religious reason

☐ 20 = Not sure
☐ 21 = Other (specify): ____

b. *[If **YES**]* Were you for or against him being circumcised before two months of age?
☐ 1 = For
☐ 0 = Against
☐ 2 = Not sure

i. Why?
*[**A:** DO NOT read list of answers –**check all that apply**. Probe ➔ Any other reason?]*
*[**B**: Read list of answers –**check all that apply**]*

A ***B***
☐ ☐ 1 = Need to consult the mother
☐ ☐ 2 = Need more information about circumcision

☐ ☐ 3 = Pain
☐ ☐ 4 = Bleeding
☐ ☐ 5 = Infection
☐ ☐ 6 = Injury to the penis
☐ ☐ 7 = Death from circumcision
☐ ☐ 8 = Going against cultural tradition
☐ ☐ 9 = It is better to wait until the boy is older
☐ ☐ 10 = The mother is against it
☐ ☐ 11 = The baby is unwell
☐ ☐ 12 = The mother is unwell / tired
☐ ☐ 13 = There is no reason to circumcise a baby boy

☐ ☐ 14 = Protection against HIV/STI
☐ ☐ 15 = Protection against Urinary Tract Infection (UTI)
☐ ☐ 16 = Penile hygiene / cleanliness
☐ ☐ 17 = Improved cosmetic appearance of the penis
☐ ☐ 18 = Less pain / the procedure is safer when done earlier
☐ ☐ 19 = Religious reason

☐ ☐ 20 = Not sure

☐ ☐ 21 = Other (specify): ____

ii. Of those reasons, which is the primary reason for your opinion about circumcision for your son? *[Read respondent's answer[s] from previous question–**check only one**]*
☐ 1 = Need to consult the mother
☐ 2 = Need more information about circumcision

☐ 3 = Pain
☐ 4 = Bleeding
☐ 5 = Infection
☐ 6 = Injury to the penis
☐ 7 = Death from circumcision
☐ 8 = Going against cultural tradition
☐ 9 = It is better to wait until the boy is older
☐ 10 = The mother is against it
☐ 11 = The baby is unwell
☐ 12 = The mother is unwell / tired
☐ 13 = There is no reason to circumcise a baby boy

☐ 14 = Protection against HIV/STI
☐ 15 = Protection against Urinary Tract Infection (UTI)
☐ 16 = Penile hygiene / cleanliness
☐ 17 = Improved cosmetic appearance of the penis
☐ 18 = Less pain / the procedure is safer when done earlier
☐ 19 = Religious reason

☐ 20 = Not sure
☐ 21 = Other (specify): ____

43. If you could do it again, would you circumcise your baby before two months of age? *[check one]*
☐ 1 = Yes
☐ 0 = No
☐ 2 = Not sure

Part 3: Beliefs about Circumcision
[Read:] Now I am going to ask you some questions about your beliefs about male circumcision

44. It easier to keep a penis clean if a man is . . . . ? *[check one]*
☐ 1 = Circumcised
☐ 2 = Uncircumcised
☐ 3 = No difference
☐ 4 = Not sure

45. It easier for a man to get a disease from a woman if the man is. . . . ? *[check one]*
☐ 1 = Circumcised
☐ 2 = Uncircumcised
☐ 3 = No difference
☐ 4 = Not sure

46. It is easier for a man to get AIDS if he is. . . . ? *[check one]*
☐ 1 = Circumcised
☐ 2 = Uncircumcised
☐ 3 = No difference
☐ 4 = Not sure

47. Men enjoy sex more if they are. . . . ? *[check one]*
☐ 1 = Circumcised
☐ 2 = Uncircumcised
☐ 3 = No difference
☐ 4 = Not sure

48. Most women enjoy sex more with a man who is. . . . ? *[check one]*
☐ 1 = Circumcised
☐ 2 = Uncircumcised
☐ 3 = No difference
☐ 4 = Not sure

49. Men are more promiscuous if they are. . . . *[check one]*
☐ 1 = Circumcised
☐ 2 = Uncircumcised
☐ 3 = No difference
☐ 4 = Not sure

50. A penis looks better if it is . . . . ? *[check one]*
☐ 1 = Circumcised
☐ 2 = Uncircumcised
☐ 3 = No difference
☐ 4 = Not sure

51. In the past month, how many shillings have you earned from all sources? *[check one]*
☐ 1 = None
☐ 2 = < 2000
☐ 3 = 2000-4999
☐ 4 = 5000-9999
☐ 5 = 10000-25000
☐ 6 = > 25000

52. Have you ever talked with the mother of the baby about her HIV status? *[check one]*
☐ 1 = Yes
☐ 0 = No
☐ 2 = Not sure
☐ 3 = Refused to answer

53. Have you ever been tested for HIV? *[check one]*
☐ 1 = Yes
☐ 0 = No
☐ 2 = Not sure
☐ 3 = Refused to answer

a. If **YES**, have you been tested in the past year? *[check one]*
☐ 1 = Yes
☐ 0 = No
☐ 2 = Not sure

☐ 3 = Refused to answer

b. If **YES**, are you willing to give your most recent results? *[check one]*
☐ 1 = Yes
☐ 0 = No

i. If **YES**, what were they? *[check one]*
☐ 1 = Positive
☐ 0 = Negative
☐ 2 = Not sure

54. Whether you have been tested or not, in your opinion, what is your HIV status? *[check one]*
☐ 1 = HIV positive
☐ 0 = HIV negative
☐ 2 = Not sure
☐ 3 = Refused to answer

55. What do you think the HIV status of the mother of the baby is? *[check one]*
☐ 1 = HIV positive
☐ 0 = HIV negative
☐ 2 = Not sure
☐ 3 = Refused to answer

56. Please provide a contact number or contact information, in case we need to ask you any follow-up questions
a. Phone number: ______________________________________

b. Phone owner (name): ______________________________________

c. Other contact information: ______________________________________

[Read:] Thank you for your time. Those are all the questions I have for you. Do you have any questions for me right now?

[To be answered by the interviewer:]

57. *Primary language of interview*
☐ 1 = English
☐ 2 = DhoLuo
☐ 3 = Kiswahili

58. *Location of interview*
☐ 1 = Participant's home
☐ 2 = Study Office
☐ 3 = Health Facility (specify): ______________________________
☐ 4 = Other (specify): ______________________________

59. *End Time*

:

60. *Interviewer code*

61. *Notes:*

____
____
____
____
____
____
